# Current practices in patient-reported outcome (PRO) data collection in clinical trials: a cross-sectional survey of UK trial staff and management

**DOI:** 10.1136/bmjopen-2016-012281

**Published:** 2016-10-03

**Authors:** Derek Kyte, Jonathan Ives, Heather Draper, Melanie Calvert

**Affiliations:** 1 Institute of Applied Health Research, University of Birmingham, Birmingham, UK; 2 School of Social and Community Medicine, University of Bristol, Bristol, UK

**Keywords:** Survey, patient-reported outcomes, PROs, patient-reported outcome measures, PROMs

## Abstract

**Objectives:**

Patient-reported outcome measures (PROMs) collected in clinical trials should be administered in a standardised way across sites and routinely screened for avoidable missing data in order to maximise data quality/minimise risk of bias. Recent qualitative findings, however, have raised concerns about the consistency of PROM administration in UK trials. The purpose of this study was to determine the generalisability of these findings across the wider community of trial personnel.

**Design:**

Online cross-sectional survey.

**Setting:**

Participants were recruited from 55 UK Clinical Research Collaboration Registered Clinical Trials Units and 19 Comprehensive Local Research Networks.

**Participants:**

Research nurses, data managers/coordinators, trial managers and chief/principal investigators involved in clinical trials collecting PROMs.

**Analysis:**

We undertook descriptive analyses of the quantitative data and directed thematic analysis of free-text comments. Factors associated with the management of missing PRO data were explored using logistic regression.

**Results:**

Survey data from 767 respondents supported the generalisability of qualitative study findings, suggesting inconsistencies in PROM administration with regard to: the level of assistance given to trial participants; the timing of PROM completion in relation to the clinical consultation; and the management of missing data. Having ≥10 years experience in a research role was significantly associated with the appropriate management of missing PROM data (OR 2.26 (95% CI 1.06 to 4.82), p=0.035). There was a consensus that more PROM guidance was needed in future trials and agreement between professional groups about the necessary components.

**Conclusions:**

There are inconsistencies in the way PROMs are administered by trial staff. Such inconsistencies may reduce the quality of data and have the potential to introduce bias. There is a need for improved guidance in future trials that support trial personnel in conducting optimal PROM data collection to inform patient care.

**Strengths and limitations of this study**

- This study is the first to survey the opinions of researchers and trial personnel regarding the administration of patient-reported outcome measures in UK clinical trials.
- The large research nurse sample size should allow generalisation of the results in this population. Owing to their much smaller sample sizes, caution should be exercised when generalising the results from the remaining subgroups.
- Respondents were self-selecting and may include those with more knowledge regarding patient-reported outcomes (PROs); this should be taken into account when interpreting the results of the study.
- As the survey was anonymised, it was not possible to link staff together on a particular study. Thus, further work is needed to definitively establish whether the PRO administration variability seen in this survey may be present in a single trial.

## Introduction

Patient-reported outcomes (PROs) are commonly measured in clinical trials in order to evaluate the effectiveness of medical interventions from the point of view of patients.^1^ PRO trial data are collected using validated questionnaires, known as patient-reported outcome measures (PROMs). PROs trial results can inform the healthcare decisions made by patients and their clinicians, support licensing claims for new medicines and influence the development of health policy, including decisions about cost-effectiveness.^2–4^ In view of their importance, there is a need to ensure rigorous PRO data collection.

Unfortunately, missing PRO data can be a problem in clinical trials. In a 2008 review of 285 randomised controlled trials (RCTs) collecting PROs, Fielding *et al*^5^ found that 18% reported between 11% and 20% missing data and a further 18% reported >20% missing data. Such data are generally considered ‘not missing at random’, rather they may be missing from those participants with the poorest outcomes, rendering it non-ignorable.^6^ Retrospective PRO data capture is frequently not possible; therefore, missing data of this type can result in bias if such participants are concentrated in a particular trial arm.^6^ ^7^

Trials should therefore be designed to ensure that PROMs are administered in a standardised way across trial sites and are routinely screened for avoidable missing data in order to maximise data quality and reduce the risk of systematic bias.^6^ ^8–10^ PROM administration guidance should, therefore, be included in the trial protocol and in site start-up training, and may also be incorporated into supporting trial documentation such as standard operating procedures (SOPs).^7^ ^11^ ^12^ Recent qualitative evidence, however, has raised concerns about the conduct of PRO measurement in UK trials.^13^ The study, conducted by the authors, outlined three main findings.^13^ First, there were reported inconsistencies in the way in which PROMs were administered that could adversely affect the quality of PRO trial data and potentially bias results. Second, there was a reported lack of PRO-specific protocol content, training and education available to trial staff. Third, data collection staff reported being intermittently exposed to PROM data that caused them to become concerned for the well-being of a trial participant (also known as a ‘PRO alert’,^14^ box 1) and, in the absence of trial level guidance, reported providing off-protocol cointerventions. Some of these interventions appeared to risk biasing the results of the trial.

Box 1 **Definitions**

Patient-reported outcome (PRO)—‘… any report of the status of a patient's health condition that comes directly from the patient, without interpretation of the patient's response by a clinician or anyone else’^4^

Patient-reported outcome measure (PROM)—A validated paper-based or electronic psychometric questionnaire used to collect PRO data

PRO alert—The exposure of data collection staff to PRO data displaying ‘concerning levels of psychological distress or physical symptoms that may require an immediate response’^14^

The aim of this study was to determine the extent to which our qualitative findings were generalisable to the wider community of trial staff using a large-scale cross-sectional national survey of UK-based trial personnel. Survey respondents’ experiences of PRO alerts and their management are presented in a separate publication.^15^ In this paper, we present the results of the survey specific to PROM administration, with the following objectives:

1. To investigate reported inconsistencies in PROM administration in trials.
2. To investigate a reported lack of PRO-specific trial protocol content and training.
3. To determine what PRO-specific trial protocol content and training respondents would like to see in future trials.

## Methods

### Survey design

An online survey (see online supplementary file 1) was developed by investigators with PRO and ethics expertise, and the content informed by the results of our qualitative study.^13^ The survey contained questions on: (1) demographics, (2) the participants’ experiences of PROM administration with reference to the most recent trial in which they had been involved, (3) the provision of PRO-specific guidance within the trial and (4) PRO guidance/training they would like to see in future trials. Most questions contained space for free-text comments, to allow respondents to expand on their answers. The survey was pilot-tested in a purposive sample of n=9 research nurses to ensure the content was clearly understood and to establish the feasibility of the distribution/collection methods. Additional free-text comment boxes were added to the survey instruments following pilot feedback. No other changes to the survey questions were necessary.

10.1136/bmjopen-2016-012281.supp1 supplementary file

### Survey sample

The study recruitment methods have been reported elsewhere.^15^ In brief, an anonymised online exploratory cross-sectional national survey of UK research nurses, data managers/coordinators, trial managers and chief and principal investigators (CPIs) involved in clinical trials using either a primary or secondary PRO was conducted in 2013/2014. The survey was anonymised to encourage respondents to freely discuss potentially controversial aspects surrounding PRO assessment/PRO alerts and to maximise responses. An email containing information about the study, and a link to the online survey, was distributed via 55 UK Clinical Research Collaboration Registered Clinical Trials Units (CRC-RCTUs) and 19 Comprehensive Local Research Networks (CLRNs). Eligible individuals were invited to click on the link and complete the survey.

### Analysis

Descriptive quantitative analysis was undertaken for each respondent group. Frequency distributions were used to describe respondent characteristics and survey responses. All analysis was conducted using SPSS (V.21, IBM). An exploratory prespecified logistic regression analysis was also undertaken to investigate which factors were associated with the appropriate management of missing PRO data by data collection staff, as this can represent an important potential source of bias. Existing literature recommends routine checking of completed PROMs and subsequent ‘chasing’ of missing data.^16^ Thus, the dependent variable in the model was the appropriate management of missing data, defined as: ‘whether the completed PROM was checked for missing data *and* participants were subsequently asked to complete missing items/questionnaires’. The independent variables were: the role of the data collector (ie, ‘research nurse’ or ‘data manager/coordinator’); their length of experience in the research role; whether PRO-specific information was reportedly present in the trial protocol; and whether PRO-specific information was reportedly included in trial training. A minimum of 60 responses were required to satisfy the sample size requirement for this regression analysis (15 per covariate).^17^ Significance was set at p<0.05.

DK undertook directed content analysis of the free-text comments responses, using the data from the qualitative study^13^ to develop the initial research questions and coding framework.^18^ Additional codes were developed as the analysis was conducted and the framework was modified accordingly.^18^ JI formally reviewed all coding to enhance trustworthiness, and any disagreements about coding were discussed and resolved.

## Results

A total of 767 individuals responded to the online survey (table 1). The respondents’ most recent experience of a trial collecting PROMs was predominantly in the secondary care setting, with trials ranging across clinical specialities (most commonly oncology). Trials appeared to use a number of different PROMs, of which the most common were the five-dimension European Quality of Life instrument (EQ-5D), Hospital Anxiety and Depression scale (HADS), the Short-Form Health Survey 12-item (SF-12) and 36-item (SF-36) questionnaires, the European Organisation for Research and Treatment of Cancer Core Quality of Life Questionnaire (EORTC QLQ-C30) and the Health Assessment Questionnaire (HAQ). The survey results are presented in table 2 and summarised below. Qualitative themes and illustrative respondent quotations are also presented. A response rate calculation is not appropriate for the study as ours was a non-probability sample.^19^ Moreover, neither the UK CRC-CTUs nor the NIHR CLRNs held data regarding the number of staff involved in trials with a primary or secondary PRO, so there was no way to determine a denominator. However, in discussion with the CRC-CTU coordinating centre and with CLRN contacts, we estimate the total number of researchers receiving our survey invite, including those individuals ineligible for the study, was ∼1800; our sample represented 43% of this number.

**Table 1:** Characteristics of respondents

Respondent characteristics (total=767)	No. (%) research nurse respondents* (n=560)	No. (%) data manager respondents* (n=41)	No. (%) trial manager respondents* (n=129)	No. (%) chief and principal investigator respondents* (n=37)
Age, years				
≤25	4 (0.7)	3 (7.9)	4 (3.1)	0 (0)
26–35	95 (17)	14 (36.8)	51 (39.5)	5 (13.5)
36–45	193 (34.5)	10 (26.3)	43 (33.3)	11 (29.7)
46–55	217 (38.8)	8 (21.1)	23 (17.8)	14 (37.8)
≥56	51 (9.1)	3 (7.9)	8 (6.2)	7 (18.9)
Years in research role				
<1	51 (9.2)	4 (10.5)	12 (9.3)	0 (0)
1–3	208 (37.3)	13 (34.2)	42 (32.6)	11 (29.7)
4–6	147 (26.4)	7 (18.4)	31 (24)	4 (10.8)
7–9	50 (9)	4 (10.5)	12 (9.3)	5 (13.5)
≥10	101 (18.1)	10 (26.3)	32 (24.8)	17 (45.9)
Setting of most recent clinical trial collecting PROs†				
Primary care	112 (20.7)	15 (39.5)	47 (37.9)	16 (44.4)
Secondary care	428 (79.3)	23 (60.5)	77 (62.1)	20 (56.6)
Clinical areas covered by most recent clinical trial collecting PROs†				
Cardiovascular	69 (16.5)	3 (9.4)	10 (10)	0 (0)
Elderly care	17 (4.1)	2 (6.3)	10 (10)	2 (7.4)
General medicine	39 (9.3)	2 (6.3)	7 (7)	0 (0)
General practice	19 (4.5)	3 (9.4)	23 (23)	9 (33.3)
Neurology	51 (12.2)	1 (3.1)	9 (9)	4 (14.8)
Obstetrics and gynaecology	22 (5.3)	3 (9.4)	7 (7)	2 (7.4)
Oncology	119 (28.5)	15 (46.9)	28 (28)	1 (3.7)
Opthalmology	8 (1.9)	1 (3.1)	4 (4)	7 (25.9)
Orthopaedics	35 (8.4)	1 (3.1)	7 (7)	1 (3.7)
Paediatrics	35 (8.4)	2 (6.3)	9 (9)	6 (22.2)
Respiratory	41 (9.8)	5 (15.6)	8 (8)	3 (11.1)
Rheumatology	47 (11.2)	1 (3.1)	6 (6)	5 (18.5)
PROs used in trial†				
EuroQol EQ-5D	401 (76.1)	25 (67.6)	99 (82.5)	24 (80)
Health Assessment Questionnaire (HAQ)	154 (29.2)	1 (2.7)	4 (3.3)	2 (6.7)
Nottingham Health Profile (NHP)	0 (0)	0 (0)	0 (0)	0 (0)
SF-12 Health Survey or SF-12v2 Health Survey	36 (6.8)	6 (16.2)	22 (18.3)	7 (23.3)
SF-36 Health Survey or SF-36v2 Health Survey	104 (19.7)	5 (13.5)	17 (14.2)	6 (20)
Hospital Anxiety and Depression scale (HAD)	115 (21.8)	4 (10.8)	21 (17.5)	11 (36.7)
Arthritis Impact Measurement Scales (AIMS2)	3 (0.6)	0 (0)	0 (0)	2 (6.7)
EORTC QLQ—C30 (Core Questionnaire)	106 (20.1)	9 (24.3)	18 (15)	0 (0)
Minnesota Living with Heart Failure Questionnaire (MLHF)	9 (1.7)	0 (0)	1 (0.8)	1 (3.3)
Oxford Hip Score (OHS)	9 (1.7)	0 (0)	0 (0)	1 (3.3)
Oxford Knee Score (OKS)	14 (2.7)	1 (2.7)	0 (0)	0 (0)
Roland-Morris Disability Questionnaire (RMDQ)	2 (0.4)	0 (0)	2 (1.7)	4 (13.3)

*Columns may not add up to n due to missing values.

†Respondents could select multiple categories.

**Table 2:** Questionnaire responses

Survey questions and response options	No. (%) research nurse respondents* (n=560)	No. (%) data manager respondents* (n=41)	No. (%) trial manager respondents* (n=129)	No. (%) chief and principal investigator respondents* (n=37)
What assistance did you give to the trial participants during the completion of the questionnaire? (Last Trial)				
I read the questions out to the participants	194 (36.9)†	–	–	–
I helped participants to understand the questions	209 (39.7)†	–	–	–
The participants gave me the answers and I filled in the questionnaire	121 (23.0)†	–	–	–
I gave no assistance, the participants filled in their questionnaires independently	348 (66.2)†	–	–	–
If the participant had to complete the quality of life or other patient-reported outcome measure questionnaire in clinic, when did they do so? (Last Trial)				
Always *before* their consultant/doctor appointment	92 (18.2)	–	–	–
Always *after* their consultant/doctor appointment	47 (9.3)	–	–	–
Variable, sometimes before and sometimes after their consultant/doctor appointment	242 (47.9)	–	–	–
Not applicable	124 (24.6)	–	–	–
Which of the following did you do after trial participants had completed their PROM? (Last Trial)				
I sent the questionnaire to the data inputting centre without looking at it	100 (19.6)†	–	–	–
I looked at the completed questionnaire to see if the participant had missed out any questions	394 (77.3)†	–	–	–
If I discovered missing items, I prompted participants to complete them	308 (60.4)†	–	–	–
I looked at the completed questionnaire to see if there were any scoring errors (eg, 2 options selected instead of 1, scoring the wrong way round, etc)	141 (27.6)†	–	–	–
If I suspected a scoring error, I prompted participants to look again at some questions, to ensure they had understood them correctly	137 (26.9)†	–	–	–
* Checked for missing PROM data and followed up participant to rectify*	277 (49.9)			
* Checked for PROM scoring errors and followed up participant to rectify*	114 (21.2)			
When the quality of life/patient-reported outcome questionnaire data were inputted, which of the following occurred? (Last Trial)				
The questionnaire was checked to see if the participant had completed all questions	–	18 (72.0)†	–	–
If items were found to be missing, trial participants were followed up in some way (eg, by post, by phone or via their research nurse) in order to complete the questionnaire	–	7 (28.0)†	–	–
The questionnaire was checked for scoring errors (eg, two answers given instead of one, or reversed scoring)	–	19 (76.0)†	–	–
If scoring errors were detected, trial participants were followed up in some way (eg, by post, by phone or via their research nurse) in order to correct them	–	4 (16.0)†	–	–
* Checked for missing PROM data and followed up participant to rectify*	–	6 (15.4)		
* Checked for PROM scoring errors and followed up participant to rectify*	–	4 (9.8)		
Were the staff involved in data collection given instructions on how to administer the quality of life/patient-reported outcome questionnaire? (Last Trial)				
Yes	–	–	90 (70.9)	29 (82.9)
No	–	–	37 (29.1)	6 (17.1)
What particular information on quality of life/patient-reported outcome measurement was given to the data collection staff in the last trial you were involved with?				
The purpose and/or importance of quality of life/patient-reported outcome data to the trial	–	–	71 (86.6)	27 (93.1)
Relevance and reasoning behind individual quality of life/patient-reported outcome questions	–	–	43 (52.4)	22 (75.9)
When to administer the questionnaire (time points)	–	–	84 (100)	29 (100)
When to administer the questionnaire during the clinic appointment (before/during/after the consultation)	–	–	53 (67.9)	22 (78.6)
How much assistance to give the participant during questionnaire completion	–	–	52 (63.4)	25 (86.2)
How to check for, and deal with, missing quality of life/patient-reported outcome data	–	–	48 (58.5)	24 (82.8)
What to do if participants write additional information on their questionnaires (or attach a letter)	–	–	23 (28.0)	12 (41.4)
Trial protocol and training questions				
The trial protocol included information about quality of life/patient-reported outcome measurement	474 (92.2)	–	–	–
* Reported PRO protocol content present and felt it was adequate for their needs*	415 (87.7)	–	–	–
I received trial training that included information on quality of life/patient-reported outcome measurement	164 (32.7)	–	–	–
* Reported receiving PRO training and felt it was adequate for their needs*	152 (94.4%)	–	–	–
The trial protocol included information about quality of life/patient-reported outcome data inputting	–	13 (50.0)	–	–
* Reported PRO protocol content present and felt it was adequate for their needs*	–	10 (76.9)	–	–
I received trial training which included information on quality of life/patient-reported outcome data inputting	–	9 (39.1)	–	–
* Reported receiving PRO training and felt it was adequate for their needs*	–	8 (88.9)	–	–
PRO assessment explanation questions				
It was explained to me why the quality of life/patient-reported outcome measure data were being collected in the trial	314 (60.5)	–	–	–
I was confident I could explain to trial participants why the quality of life/patient-reported outcome measure data were being collected in the trial	456 (87.7)	–	–	–
It was explained to me why each of the questions in the quality of life/patient-reported outcome measure were included, ie, how each was of relevance to the trial	157 (30.3)	–	–	–
I was confident I could explain to trial participants why each of the questions in the quality of life/patient-reported outcome measure had been included, ie, how each was of relevance to the trial	312 (59.9)	–	–	–
Please read the following statements. In each case, please indicate whether you ‘strongly agree’, ‘agree’, have ‘no opinion’, ‘disagree’ or ‘strongly disagree’ with the statement (Future Trials)				
There should be more protocol content and trial training covering quality of life/patient-reported outcome measurement, in trials employing such outcomes	SA 140 (27.9) A 283 (56.5) NO 57 (11.4) D 20 (4.0) SD 1 (0.2)	–	–	–
There should be more quality of life/patient-reported outcome measurement guidance contained within other trial documentation, such as site manuals or standard operating procedures, in trials employing such outcomes	SA 127 (25.4) A 302 (60.4) NO 52 (10.4) D 18 (3.6) SD 1 (0.2)	–	–	–
Please read the following statements. In each case, please indicate whether you ‘strongly agree’, ‘agree’, have ‘no opinion’, ‘disagree’ or ‘strongly disagree’ with the statement (Future Trials)				
There should be more protocol content and trial training for data managers/inputters, covering quality of life/patient-reported outcome measurement	–	SA 3 (10.7) A 17 (60.7) NO 2 (7.1) D 6 (21.4) SD 0 (0)	–	–
There should be site manuals or standard operating procedures available to data mangers/inputters that include information on quality of life/patient-reported outcome administration in the trial	–	SA 6 (21.4) A 18 (64.3) NO 3 (10.7) D 1 (3.6) SD 0 (0)	–	–
Please read the following statements. In each case, please indicate whether you ‘strongly agree’ (SA), ‘agree’ (A), have ‘no opinion’ (NO), ‘disagree’ (D) or ‘strongly disagree’ (SD) with the statement (Future Trials)				
Data collection staff in trials need more information on quality of life/patient-reported outcome measurement—in the trial protocol	–	–	SA 17 (14.8) A 33 (28.7) NO 23 (20.0) D 39 (33.9) SD 3 (2.6)	SA 6 (16.7) A 12 (33.3) NO 12 (33.3) D 5 (13.9) SD 1 (2.8)
Data collection staff in trials need more information on quality of life/patient-reported outcome measurement—in other trial documentation, such as SOPs	–	–	SA 17 (14.8) A 54 (47.0) NO 19 (16.5) D 22 (19.1) SD 3 (2.6)	SA 7 (19.4) A 16 (44.4) NO 8 (22.2) D 4 (11.1) SD 1 (2.8)
Data collection staff in trials need more information on quality of life/patient-reported outcome measurement—delivered in the form of trial training	–	–	SA 24 (21.1) A 55 (48.2) NO 17 (14.9) D 17 (14.9) SD 1 (0.9)	SA 6 (16.7) A 14 (38.9) NO 72 (19.4) D 8 (22.2) SD 1 (2.8)
It is important to explain to data collection staff, the purpose and importance of quality of life/patient-reported outcome data to the trial	–	–	SA 41 (36.3) A 69 (61.1) NO 3 (2.7) D 0 (0) SD 0 (0)	SA 13 (33.3) A 20 (55.6) NO 2 (5.6) D 1 (2.8) SD 1 (2.8)
It is important to explain to data collection staff, the relevance and reasoning behind individual quality of life/patient-reported outcome questions	–	–	SA 30 (26.1) A 55 (47.8) NO 18 (15.7) D 12 (10.4) SD 0 (0)	SA 8 (22.2) A 22 (61.1) NO 4 (11.1) D 0 (0) SD 2 (5.6)
Thinking about the future. What particular quality of life/patient-reported outcome guidance should be included the trial protocol, what should be included in trial training, and what should be included in a standard operating procedure? (TP, trial protocol; TT, trial training; SOP, standard operating procedure)				
Purpose/importance of quality of life/patient-reported outcome data in trial	TP 389 (79.1) TT 344 (69.9) SOP 131 (26.6)	–	TP 77 (67.5) TT 89 (78.1) SOP 28 (24.6)	TP 27 (87.1) TT 23 (74.2) SOP 15 (48.4)
How to administer the questionnaire	TP 212 (43.1) TT 403 (81.9) SOP 275 (55.9)	–	TP 43 (38.1) TT 101 (89.4) SOP 73 (64.6)	TP 13 (40.6) TT 27 (84.4) SOP 23 (71.9)
How to input quality of life/patient-reported outcome data into the database‡	–	TP 3 (11.1) TT 22 (81.5) SOP 18 (66.7)	–	–
When to administer the questionnaire	TP 359 (73.9) TT 328 (67.5) SOP 202 (41.6)	–	TP 88 (77.2) TT 95 (83.3) SOP 64 (56.1)	TP 23 (71.9) TT 28 (87.5) SOP 21 (65.6)
What to do if there are missing data or in the event of scoring errors (eg, two answers provided instead of one, or reversed scoring)‡	–	TP 2 (7.3) TT 19 (67.3) SOP 28 (78.2)	–	–
What to do if participants write additional information on their questionnaires (or attach a letter)	TP 178 (36.3) TT 405 (82.5) SOP 232 (47.3)	TP 3 (10.7) TT 20 (71.4) SOP 28 (64.3)	TP 14 (12.7) TT 83 (75.5) SOP 78 (70.9)	TP 5 (15.6) TT 25 (78.1) SOP 22 (68.8)
Ethical issues associated with quality of life/patient-reported outcome use	TP 253 (52.5) TT 345 (71.6) SOP 180 (37.3)	–	TP 57 (56.4) TT 68 (67.3) SOP 36 (35.6)	TP 12 (40.0) TT 24 (80.0) SOP 16 (53.3)
How to deal with upset patients (communication/counselling skills)	TP 71 (15.2) TT 390 (83.7) SOP 204 (43.8)	–	TP 6 (6.0) TT 91 (91.0) SOP 50 (50.0)	TP 6 (18.8) TT 29 (90.6) SOP 17 (53.1)
Working with non-English language patients	TP 248 (51.8) TT 329 (68.7) SOP 284 (59.3)	–	TP 39 (38.2) TT 80 (78.4) SOP 66 (64.7)	TP 18 (58.1) TT 24 (77.4) SOP 20 (64.5)
How to support the participant to answer sensitive questions	TP 76 (15.9) TT 429 (89.7) SOP 180 (37.7)	–	TP 4 (3.7) TT 100 (92.6) SOP 45 (41.7)	TP 8 (27.6) TT 27 (93.1) SOP 19 (65.5)
How to collect quality of life/patient-reported outcome data without biasing the results	TP 190 (38.7) TT 412 (83.9) SOP 265 (54.0)	–	TP 32 (28.6) TT 98 (87.5) SOP 63 (56.3)	TP 9 (28.1) TT 29 (90.6) SOP 21 (65.1)
Collecting quality of life/patient-reported outcome data in different patient groups and/or settings	TP 145 (30.3) TT 381 (79.9) SOP 220 (46.0)	–	TP 24 (25.0) TT 75 (78.1) SOP 42 (43.8)	TP 12 (41.4) TT 23 (79.3) SOP 16 (55.2)
Relevance and reasoning behind individual quality of life/patient-reported outcome questions	TP 269 (55.1) TT 371 (76.0) SOP 94 (19.3)	–	TP 50 (54.3) TT 66 (71.7) SOP 17 (18.5)	TP 12 (42.9) TT 23 (82.1) SOP 10 (35.7)
How to deal with difficult situations.	TP 71 (15.2) TT 391 (83.7) SOP 94 (45.6)	–	TP 2 (2.0) TT 88 (88.9) SOP 41 (41.4)	TP 5 (16.7) TT 27 (90.0) SOP 22 (73.3)

A, agree; D, disagree; N, no; NO, no opinion; SA, strongly agree; SD, strongly disagree; SOP, standard operating procedure; TP, trial protocol; TT, trial training; Y, yes.

*Columns may not add up to n due to missing values.

†Respondents were able to select multiple categories. Full survey data set available in online supplementary file 2.

‡Data manager respondents only.

10.1136/bmjopen-2016-012281.supp2 supplementary file

### Inconsistencies in PROM administration

#### Participant assistance

Respondents were asked what level of assistance they had given to participants completing PROMs during their most recent relevant trial. 66.2% of research nurse respondents reported giving no assistance. The remainder gave assistance in a variety of different ways. Of these, 37.9% reported helping participants to understand the questions, 36.9% reported reading the PROM questions out to the participants and 23.0% reported being given the answers by participants then filling in the questionnaire on their behalf.

#### Timing of PROM completion

There were varying responses with regard to the timing of questionnaire completion. 47.9% of nurses reported that the timing with which they administered the PROM varied (ie, it was ‘sometimes before’ and ‘sometimes after’ the clinical consultation) during their most recent trial. 9.3% reported routinely administering the PROM *after* the consultation. Only 18.2% reported routinely administering PROMs *prior* to the participant's clinical consultation, inline with suggested guidelines.^20^ ^21^

#### Management of missing PRO data

77.3% of research nurses and 72.0% of data managers reported routinely checking completed PROM questionnaires for missing data in their most recent trial. However, of the total sample, only 49.9% research nurses and 15.4% of data managers checked for missing data *and* subsequently attempted to follow-up participants to complete the missing items. 27.6% of research nurses and 76.0% of data mangers reported checking PROM question responses for scoring errors (eg, where two options were selected instead of one), which are often logged as missing data. Of the total sample, just 21.2% research nurses and 9.8% of data managers reported checking for scoring errors and subsequently attempting to follow-up participants in order to correct the errors.

#### Determinants of differences in the management of missing data

The findings of the exploratory logistic regression analysis are available in online supplementary file 3. In the final model, only ‘10 years or more experience in the research role’ was significant (p=0.035). The odds of individuals with such experience routinely checking *and* chasing missing PROM data were 2.26 (95% CI 1.06 to 4.82) times higher than those with less experience. There were no significant associations between the dependent variable and ‘the role of the data collector’ (p=0.45); ‘whether PRO-specific information was reportedly provided in the trial protocol’ (p=0.94); or ‘whether PRO-specific information was reportedly included in trial training’ (p=0.64).

10.1136/bmjopen-2016-012281.supp3 supplementary file

### Current PRO-specific protocol content and training

Survey respondents were questioned about the PRO-specific protocol content and training delivered in their most recent trial. Results are presented first from members of the trial management team (CPIs and trial managers), and then from frontline data collection staff (research nurses and data managers/coordinators).

#### Protocol content and training provision

82.9% of CPIs and 70.9% of trial managers reported giving instructions to trial staff on how to administer the PROM questionnaire in their most recent relevant trial. Respondents were asked what particular information was provided to data collection staff. 93.1% of CPIs and 86.6% of trial managers reported providing information about the purpose and/or importance of PROM data to the trial and 75.9% and 52.4%, respectively, reported giving information surrounding the relevance and reasoning behind individual PROM questions. One hundred per cent of respondents in both groups reported providing information on when to administer the PROM questionnaire. Over three-quarters of CPIs reported providing information on when to administer the PROM during a clinic (78.6%); how much assistance to give the participant during questionnaire completion (86.2%); and how to check for, and deal with, missing PRO data (82.8%). The proportion of trial managers who reported providing this information was uniformly lower: 67.9%, 63.4% and 58.5%, respectively. 41.4% of CPIs and 28.0% of trial managers reported providing information on what action should be taken if participants had written additional information on their PROM, or had attached further information (such as letter to the trial team) to their PROM.

#### Protocol content and training available to frontline staff

92.2% of research nurses and 50.0% of data managers/coordinators reported that the trial protocol had included some form of PRO-specific information, with 87.7% and 76.9%, respectively, reporting that the content was adequate for their needs. 32.7% of research nurses and 39.1% of data managers/coordinators reported receiving trial training that incorporated PRO guidance. 94.4% of research nurses and 88.9% of data managers/coordinators who reported receiving PRO training felt it was adequate for their needs. 60.5% of research nurses reported they had received an explanation of why the PROM was being collected in the trial and 87.7% felt confident they could explain this to participants. 30.3% of research nurses reported receiving an explanation regarding the relevance and reasoning behind individual PROM questions and 59.9% felt confident they could explain this to their participants.

#### Free-text comments relating to trial protocol content and training

There were 40 free-text comments in this section, with most appearing to suggest that, while nurses felt PRO-specific information was generally present within trial protocols, it could be limited in depth:

Usually there is reference to the fact that the questionnaires are to be completed. No other information is provided or instructions on use, administration etc. (Research nurse)

A number of comments suggested that nursing staff had received little in the way of PRO training:

I was not given any training on PROM basically just been told if patients consent for the study they fill this document. (Research nurse)

Some research nurses suggested PROM training was particularly lacking for staff joining the trial at a later stage:

Our centre was invited to take part in the study quite late on so missed the initial set up that other centres had. Whilst there was some verbal communication regarding how to deliver the questionnaires much of it was down to previous experience/personal communication skills…. (Research nurse)

I took over the study partway through and received minimal instruction relating to the questionnaires. Anything additional I learn en route. (Research nurse)

Some reported that PRO trial training was inconsistently delivered across trials:

I have been taught how to use it many times but not for this study. However, to ensure consistency I believe we should be trained on this for each study. (Research nurse)

A number of comments implied that the impact of a lack of guidance may be minimal. These nurses reported either relying on either previous experience of PRO assessment in trials, or knowledge gained via attendance at previous training courses, or an independent search for the information they required:

I have used QOL questionnaires a fair amount so felt confident using the provided tools without needing training. (Research nurse)

No specific training given by the study centre for this study, but I have completed training on many of the QoL [PROMs] previously. (Research nurse)

The trial training said that the questionnaires had to be done by the patient, but did not give any reasons why. I did my own reading to find out why this was the case. (Research nurse)

Conversely, one respondent comment suggested a lack of PROM guidance resulted in an impaired ability to explain aspects surrounding PROM assessment to trial participants:

I can roughly explain to participants why this information is required, but would prefer to have a better understanding myself to be able to fully explain this to study participants. (Research nurse)

and one respondent comment suggested it led to more queries to the trial team:

The trial coordinator had to be contacted quite often for clarification as subject asked questions that was not covered in the training session. (Research nurse)

### Future PRO-specific protocol content and training

Survey respondents were asked whether they felt more PRO guidance was needed in future trials. While 85.1% of research nurses and 78.6% of data managers/coordinators ‘strongly agreed’ or ‘agreed’, there should be more PRO guidance provided in future trials with PRO end points, in contrast, only 58.2% of trial managers and 56.5% of CPIs felt the same.

After considering a list of possibilities suggested by the findings of our qualitative study, respondents were also asked which particular PRO-specific items of information they felt were needed and where should they be provided: in the trial protocol, in trial training or in supporting trial documentation such as SOPs. In general, all groups supported the inclusion of the majority of proposed PRO guidance within trial training and/or a SOP, but there was less support for including items within the protocol. In order to highlight where there was agreement on the necessary components of PRO guidance, items that were selected by more than 50% of respondents in a professional group are presented in table 3.

**Table 3:** Future patient-reported outcome guidance provision

	Trial protocol				Trial training				SOP			
Suggested PRO-specific items of information^13^	RN	DM	TM	CPI	RN	DM	TM	CPI	RN	DM	TM	CPI
Purpose/importance of quality of life/patient-reported outcome data in trial	*		*	*	*		*	*				
How to administer the questionnaire					*		*	*	*		*	*
How to input quality of life/patient-reported outcome data into the database						*				*		
When to administer the questionnaire	*		*	*	*		*	*			*	*
What to do if there is missing data or in the event of scoring errors (eg, two answers provided instead of one, or reversed scoring)						*				*		
What to do if participants write additional information on their questionnaires (or attach a letter)					*	*	*	*		*	*	*
Ethical issues associated with quality of life/patient-reported outcome use	*				*		*	*				
How to deal with upset patients					*		*	*				*
Working with non-English language patients	*			*	*		*	*	*		*	*
How to support the participant to answer sensitive questions					*		*	*				*
How to collect quality of life/patient-reported outcome data without biasing the results					*		*	*	*		*	*
Collecting quality of life/patient-reported outcome data in different patient groups and/or settings					*		*	*				
Relevance and reasoning behind individual quality of life/patient-reported outcome questions	*				*		*	*				
How to deal with difficult situations					*		*	*				*

#### Free-text comments regarding future PRO guidance

There were 22 free-text comments in this section. These most commonly suggested that trial protocols should signpost sources of PRO information, rather than include the information themselves:

I think that mention of some things within the Protocol could be quite short e.g. ‘how to deal with difficult situations will be covered in trial training and in SOP xxx date yyy’. (Research nurse)

Some comments indicated PRO training should be conducted outside of the trial:

some of this could be generic to many trials so may be able to train as ‘general training’ via R&D depts rather than trial specific training via CTUs. (Research nurse)

Two research nurses each suggested one additional element of PRO guidance, but the optimal location was not specified:

whether the questionnaire should always be answered by the individual or whether it can be used by family/friends on the patient's behalf. (Research nurse)

how to answer when a question is ambiguous or the [information] given does not fit in with the suggested answer. (Research nurse)

Finally, one research nurse highlighted the importance of including PROM guidance in the participant information:

You haven't asked about putting this into the [participant] info sheets which is very important. The patients need to know exactly what is required of them…. (Research nurse)

## Discussion

### Principal findings

The survey findings support the generalisability of our previous qualitative evidence,^13^ suggesting there are inconsistencies in the way PROMs are administered by trial personnel, with regard to: the level of assistance given to participants during PROM completion; the timing with which PROMs are completed in relation to the clinical consultation; and the way missing PRO data are monitored and acted on.

This variability in PRO administration practice is problematic on two fronts. Where it exists between trials, it may lessen the confidence with which different PRO trial results may be compared by key stakeholders, including: patients, clinicians, regulatory authorities and policymakers. Where variability is present in a single trial, however, it raises a number of concerns. First, marked differences reported in the level of assistance given to trial participants during PRO assessment may result in measurement variability within the study, reducing the quality of the trial data. In addition, increased assistance given to some participants could lead to response bias.^22^ Second, the practice of administering PROM questionnaires after a clinical consultation may lead to PRO data contamination, as, if a participant receives bad news or undergoes an invasive procedure, this may colour their questionnaire responses.^20^ Our data suggest that the timing of questionnaire delivery may not be consistent between individual trial staff; therefore, it may not be possible to compensate in the analyses for this potential confounder. Third, variation seen in the management of missing PRO data risks introducing bias, as data are more likely to be missing from those participants in a trial with the poorest outcomes,^6–8^ ^10^ who may be concentrated in a particular arm of the trial, for example, if one intervention in the study results in greater levels of side effects or toxicity.^23^ In our sample, over one-fifth of research nurses and data manager respondents reported that they did not check completed PROMs for missing data. In addition, only 50% of research nurses and 15% of data managers/coordinators who did report checking, subsequently followed up with participants to ensure the missing items or questionnaires were completed. It is concerning that a sizeable proportion of staff did not routinely check for missing PRO data; however, the low rates of follow-up across all data collection personnel are equally worrying: there is little point in monitoring missing data if nothing is performed to rectify the situation. These findings suggest that a formal procedure needs to be in place for monitoring *and* responding to missing PRO items/questionnaires in trials. Communicating this procedure to all frontline staff may help prevent the relatively high rates of missing PRO data seen in some studies.^5^ Failure to standardise PRO assessment methods and minimise avoidable missing data reduces data quality, misuses valuable participant time and research resources, risks the introduction of bias and ultimately devalues these important patient-centred outcomes, undermining their usefulness in informing policy, regulatory decisions and patient care, resulting in ‘research waste’.^6^ ^24^

Our previous qualitative data suggested a lack of PRO-specific guidance in the trial protocol and in the form of trial training.^13^ The survey findings revealed mixed opinions in this area. Over two-thirds of trial management respondents reported giving instructions to data collection staff on how to administer the PROM questionnaire. Research nurses concurred that PRO information was commonly present in the protocol, whereas only half of data managers agreed. The research nurse reports are, however, at odds with other available evidence. A recent review of the PRO content of trial protocols^25^ found that instructions on the PRO rationale/hypothesis, data collection methods, training and management were frequently absent from the protocols, even when the PRO was the primary outcome. Research nurse free-text comments in the survey appeared to align with these findings, suggesting that PRO protocol content could often consist of little more than a statement outlining that a PROM would be collected.

There was, however, some agreement between staff groups regarding levels of PRO training. Less than one-third of research nurses and less than two-fifths of data managers reported receiving trial training that incorporated PRO guidance. Moreover, some staff reported PRO-specific training provision was inconsistent across trials and that training was not generally accessible for staff entering a trial once it was underway.

Some research nurses in our survey questioned the usefulness of PRO-specific trial guidance, appearing to rely on their experience and judgement instead. Also, our regression model indicated that staff with greater experience (≥10 years) tended to report dealing more appropriately with missing PRO data, presumably because they had been involved in a greater number of relevant trials, but that trial protocol content or training were not significant predictors. It is possible, however, that the protocol content and training received by the respondents did not contain adequate information on the management of missing data. A review of PRO protocol content^25^ reported that under half of the n=75 included protocols detailed plans to minimise levels of avoidable missing PRO data.

When asked about future trials, an overwhelming majority of research nurse and data manager respondents reported that more PRO information should be provided in trials. It would appear, however, that respondents did not feel additional PRO-specific content in protocols was useful and that other mediums of information transfer, including trial training and SOPs, might be more appropriate. Views differed regarding the exact PRO information that should be provided to each professional group, indicating that each had different needs. This should be taken into account during the design of future trials collecting PROMs.

The findings of this study suggest there is a need to develop general PRO guidance for trialists, which highlight the key issues that should be considered by researchers involved in trial design and implementation. The study team are currently working to produce such guidelines in partnership with the International Society for Quality of Life Research ‘Best Practices for PROs in Randomised Clinical Trials’ taskforce.

### Strengths and limitations of the study

This study is the first to survey the opinions of researchers and trial personnel regarding the administration of PROs in trials. Our large research nurse sample size should allow reasonably confident generalisation of the results in this population. Owing to their much smaller sample sizes, caution should be exercised when generalising the results from the remaining subgroups. Respondents were self-selecting and may include those with more knowledge regarding PROs; this should be taken into account when interpreting the results of the study. As the survey was anonymised, it was not possible to link staff together within a particular study or study centre. We were therefore unable to determine to what extent the broader population of UK study centres was represented in the survey. In addition, further work is needed to definitively establish whether the PRO administration variability seen in this survey may be present in a single trial.

## Conclusions

The findings of this large-scale survey of clinical trial staff suggest there are inconsistencies in the way PROMs are administered by trial staff, which may reduce the quality of PRO data and have the potential to introduce bias. There was general agreement among respondents that the provision of PRO guidance in future trials should be improved, with the majority of information included in trial training and SOPs, and signposted in the trial protocol.
