# Supplementary material for: Current practices in patient-reported outcome (PRO) data collection in clinical trials: a cross-sectional survey of UK trial staff and management
Source: BMJ Open. 2016 Oct 3;6(10):e012281. doi: 10.1136/bmjopen-2016-012281 (PMC5073494; doi:10.1136/bmjopen-2016-012281)
Supplement: supplementary file [file bmjopen-2016-012281supp3.pdf]

Supplementary File 3 - Logistic Regression Results

					95% CI for OR	
	**Coefficient**	**SE**	**P**	**OR**	**Lower**	**Upper**
**Constant**	-0.889	0.961				
≥10 years experience as a research nurse	0.814	0.386	0.035	2.258	1.059	4.815

CI, confidence interval; OR, odds ratio, SE, standard error. (Full model presented in below)

Appendix 2

Full Logistic Regression Model

Variables in the Equation

		B	S.E.	Wald	df	Sig.	Exp(B)	95% C.I.for EXP(B)	
								Lower	Upper
Step 1[a]	Research_Role(1)	.667	.879	.575	1	.448	1.948	.348	10.918
	Research_Experience			5.659	4	.226			
	Research_Experience(1)	-.442	1.227	.130	1	.719	.643	.058	7.122
	Research_Experience(2)	-20.314	28420.777	.000	1	.999	.000	.000	.
	Research_Experience(3)	-20.099	40193.049	.000	1	1.000	.000	.000	.
	Research_Experience(4)	.814	.386	4.444	1	.035	2.258	1.059	4.815
	Trial_Protocol(1)	.028	.347	.007	1	.935	1.029	.521	2.032
	Trial_Training(1)	.617	1.315	.220	1	.639	1.854	.141	24.405
	Research_Experience * Research_Role			1.000	3	.801			
	Research_Experience(1) by Research_Role(1)	1.190	1.190	1.000	1	.317	3.286	.319	33.826
	Research_Experience(2) by Research_Role(1)	20.623	28420.777	.000	1	.999	904623446	.000	.
	Research_Experience(3) by Research_Role(1)	20.592	40193.049	.000	1	1.000	876813138	.000	.
	Trial_Protocol(1) by Trial_Training(1)	-.860	1.332	.417	1	.519	.423	.031	5.759
	Constant	-.889	.961	.855	1	.355	.411		

a. Variable(s) entered on step 1: Research_Role, Research_Experience, Trial_Protocol, Trial_Training, Research_Experience * Research_Role , Trial_Protocol * Trial_Training .

Key: Research_Role, Research_Experience, number of years of research experience (1=0 to 3 years, 2=4 to 6 years, 3=7 to 10 years, 4= 10+ years); Trial_Protocol, whether PRO-specific information was reportedly included in the trial protocol (1=yes); Trial_Training, whether PRO-specific information was reportedly included in trial training (1=yes)
