# Supplementary material for: Current practices in patient-reported outcome (PRO) data collection in clinical trials: a cross-sectional survey of UK trial staff and management
Source: BMJ Open. 2016 Oct 3;6(10):e012281. doi: 10.1136/bmjopen-2016-012281 (PMC5073494; doi:10.1136/bmjopen-2016-012281)
Supplement: supplementary file [file bmjopen-2016-012281supp2.pdf]

ID	First, some questions about yourself. How long have you been qualified as a nurse?	How long have you worked as a research nurse in total?	Thinking about the <u>last</u> trial you worked on that used a Quality of Life or other Patient-Reported Outcome measure. Were you employed as a research nurse in primary care or secondary care?	Thinking about the <u>last</u> trial you worked on that used a Quality of Life or other Patient-Reported Outcome measure. Were you employed as a research nurse in primary care or secondary care? - Other (please specify)	Which of the following clinical areas did the trial cover? PLEASE TICK ALL THAT APPLY - General Practice	Which of the following clinical areas did the trial cover? PLEASE TICK ALL THAT APPLY - Orthopaedics	Which of the following clinical areas did the trial cover? PLEASE TICK ALL THAT APPLY - General Medicine	Which of the following clinical areas did the trial cover? PLEASE TICK ALL THAT APPLY - Rheumatology	Which of the following clinical areas did the trial cover? PLEASE TICK ALL THAT APPLY - Cardiovascular	Which of the following clinical areas did the trial cover? PLEASE TICK ALL THAT APPLY - Oncology	Which of the following clinical areas did the trial cover? PLEASE TICK ALL THAT APPLY - Elderly Care	Which of the following clinical areas did the trial cover? PLEASE TICK ALL THAT APPLY - Respiratory	Which of the following clinical areas did the trial cover? PLEASE TICK ALL THAT APPLY - Opthalmology	Which of the following clinical areas did the trial cover? PLEASE TICK ALL THAT APPLY - Obstetrics & Gynaecology	Which of the following clinical areas did the trial cover? PLEASE TICK ALL THAT APPLY - Paediatrics	Which of the following clinical areas did the trial cover? PLEASE TICK ALL THAT APPLY - Neurology	Which of the following clinical areas did the trial cover? PLEASE TICK ALL THAT APPLY - Other(s) (please specify)	Which of the following Patient-Reported Outcome Measures did the trial use? PLEASE TICK ALL THAT APPLY - Euroqol EQ-5D	Which of the following Patient-Reported Outcome Measures did the trial use? PLEASE TICK ALL THAT APPLY - Health Assessment Questionnaire (HAQ)	Which of the following Patient-Reported Outcome Measures did the trial use? PLEASE TICK ALL THAT APPLY - Nottingham Health Profile (NHP)	Which of the following Patient-Reported Outcome Measures did the trial use? PLEASE TICK ALL THAT APPLY - SF-12® Health Survey <u>or</u> SF-12v2™ Health Survey	Which of the following Patient-Reported Outcome Measures did the trial use? PLEASE TICK ALL THAT APPLY - SF-36® Health Survey <u>or</u> SF-36v2™ Health Survey	Which of the following Patient-Reported Outcome Measures did the trial use? PLEASE TICK ALL THAT APPLY - Hospital Anxiety and Depression scale (HAD)	Which of the following Patient-Reported Outcome Measures did the trial use? PLEASE TICK ALL THAT APPLY - Arthritis Impact Measurement Scales (AIMS2)	Which of the following Patient-Reported Outcome Measures did the trial use? PLEASE TICK ALL THAT APPLY - EORTC QLQ - C30 (Core Questionnaire)	Which of the following Patient-Reported Outcome Measures did the trial use? PLEASE TICK ALL THAT APPLY - Minnesota Living with Heart Failure © Questionnaire (MLHF)	Which of the following Patient-Reported Outcome Measures did the trial use? PLEASE TICK ALL THAT APPLY - Oxford Hip Score (OHS)	Which of the following Patient-Reported Outcome Measures did the trial use? PLEASE TICK ALL THAT APPLY - Oxford Knee Score (OKS)	Which of the following Patient-Reported Outcome Measures did the trial use? PLEASE TICK ALL THAT APPLY - Roland-Morris Disability Questionnaire (RMDQ)	Which of the following Patient-Reported Outcome Measures did the trial use? PLEASE TICK ALL THAT APPLY - Don't know	Which of the following Patient-Reported Outcome Measures did the trial use? PLEASE TICK ALL THAT APPLY - Can't remember	Thinking about the <u>last</u> trial you worked on that used a Quality of Life or other Patient-Reported Outcome measure. What assistance did you give to the trial participants during the completion of the questionnaire? PLEASE TICK ALL THAT APPLY - I read the questions out to the participants.	Thinking about the <u>last</u> trial you worked on that used a Quality of Life or other Patient-Reported Outcome measure. <p>What assistance did you give to the trial participants during the completion of the questionnaire? PLEASE TICK ALL [illegible]	Thinking about the <u>last</u> trial you worked on that used a Quality of Life or other Patient-Reported Outcome measure. <p>What assistance did you give to the trial participants during the completion of the questionnaire? PLEASE TICK ALL [illegible]
1	10 years o	1-3 years	Secondary Care													Neurology		Euroqol EQ	Health Assessment Qu		SF-12® Health Survey		Hospital Anxiety and Depression scale (HAD)											
2	10 years o	1-3 years	Secondary Care														Dematolog	Euroqol EQ	Health Assessment Questionnaire (HAQ)													I read the questions out t	I helped pa	The partic
3	10 years o	10 years or more	Secondary Care							Oncology															EORTC QLQ - C30 (Core Questionnaire)									
4	7-9 years	1-3 years	Secondary Care														Gastroente	Euroqol EQ-5D																
5	10 years o	1-3 years	Secondary Care						Cardiovascular								stroke and	Euroqol EQ-5D														I read the questions out t	I helped pa	The partic
6	10 years o	1-3 years	Secondary Care							Oncology															EORTC QLQ - C30 (Core Questionnaire)									
7	10 years o	7-9 years	Secondary Care							Oncology								Euroqol EQ	Health Assessment Questionnaire (HAQ)				Hospital Anxiety and D		EORTC QLQ - C30 (Core Questionnaire)								I helped participants	
8	10 years o	Less than 1 year	Secondary Care						Cardiovascular									Euroqol EQ-5D														I read the questions out to the partic		The partic
9	10 years o	1-3 years	Secondary Care														stroke	Euroqol EQ	Health Assessment Questionnaire (HAQ)				Hospital Anxiety and Depression scale (HAD)									I read the questions out to the partic		The partic
10	7-9 years	1-3 years	Secondary Care														Stroke	Euroqol EQ-5D				SF-36® Hea	Hospital Anxiety and Depression scale (HAD)									I read the questions out t	I helped pa	The partic
11	10 years o	1-3 years	Secondary Care														renal	Euroqol EQ-5D																
12	10 years o	Less than 1 year	Secondary Care					Rheumatology																										
13	10 years o	4-6 years	Secondary Care														renal resea	Euroqol EQ-5D																
14	10 years o	Less than 1 year	Secondary Care						Cardiovascular									Euroqol EQ-5D														I read the questions out t	I helped pa	The partic
15	10 years o	Less than 1 year	Primary Care												Paediatrics				Health Assessment Questionnaire (HAQ)														I helped participants	
16	10 years o	4-6 years	Secondary Care			Orthopaedics												Euroqol EQ-5D										Oxford Knee Score (OKS)						The partic
17	7-9 years	1-3 years	Secondary Care														Stroke	Euroqol EQ	Health Assessment Questionnaire (HAQ)				Hospital Anxiety and Depression scale (HAD)									I read the questions out t	I helped participants	
18	10 years o	1-3 years	Secondary Care						Cardiovascular									Euroqol EQ-5D																
19	10 years o	1-3 years	Secondary Care												Paediatrics																			
20	10 years o	7-9 years	Secondary Care							Oncology								Euroqol EQ-5D							EORTC QLQ - C30 (Core Questionnaire)									
21	7-9 years	Less than 1 year	Secondary Care							Oncology							HAEMATO	Euroqol EQ-5D							EORTC QLQ - C30 (Core Questionnaire)									
22	10 years o	10 years or more	Secondary Care						Cardiovascular									Euroqol EQ-5D																
23	10 years o	10 years or more	Secondary Care										Opthalmology					Euroqol EQ	Health Assessment Questionnaire (HAQ)													I read the questions out t	I helped pa	The partic
24	7-9 years	4-6 years	Secondary Care														Dermatolo	Euroqol EQ	Health Assessment Questionnaire (HAQ)													I read the questions out t	I helped participants	
25	10 years o	4-6 years								Oncology								Euroqol EQ-5D							EORTC QLQ - C30 (Core Questionnaire)							I read the questions out t	I helped pa	The partic
26	10 years o	7-9 years	Secondary Care							Oncology							urology	Euroqol EQ-5D																
27	10 years o	1-3 years	Secondary Care												Paediatrics			Euroqol EQ-5D															I helped participants	
28	7-9 years	4-6 years	Secondary Care														stroke	Euroqol EQ	Health Assessment Questionnaire (HAQ)				Hospital Anxiety and Depression scale (HAD)									I read the questions out t	I helped pa	The partic
29	10 years o	10 years or more	Secondary Care		General Practice				Cardiovascular			Respiratory	Opthalmology			Neurology		Euroqol EQ-5D			SF-12® He	SF-36® Health Survey <u>or</u> SF-36v2™ Health Survey												
30	10 years o	10 years or more	Secondary Care							Oncology													Hospital Anxiety and D		EORTC QLQ - C30 (Core Questionnaire)									
31	10 years o	1-3 years	Secondary Care						Cardiovascular									Euroqol EQ-5D				SF-36® Health Survey <u>or</u> SF-36v2™ Health Survey												
32	10 years o	7-9 years	Secondary Care						Cardiovascular		Elderly Care					Neurology		Euroqol EQ	Health Assessment Questionnaire (HAQ)				Hospital Anxiety and Depression scale (HAD)									I read the questions out t	I helped pa	The partic
33	10 years o	7-9 years	Secondary Care												Paediatrics			Euroqol EQ-5D																
34	10 years o	1-3 years	Secondary Care						Cardiovascular									Euroqol EQ-5D														I read the questions out to the partic		The partic
35	10 years o	1-3 years	Secondary Care				General Medicine											Euroqol EQ-5D				SF-36® Health Survey <u>or</u> SF-36v2™ Health Survey										I read the questions out t	I helped pa	The partic
36	10 years o	1-3 years	Secondary Care							Oncology								Euroqol EQ-5D							EORTC QLQ - C30 (Core Questionnaire)								I helped participants	
37	10 years o	10 years or more	Secondary Care														hepatolog	Euroqol EQ	Health Assessment Questionnaire			SF-36® Health Survey <u>or</u> SF-36v2™ Health Survey												
38	10 years o	10 years or more	Secondary Care													Neurology			Health Assessment Questionnaire			SF-36® Health Survey <u>or</u> SF-36v2™ Health Survey												
39	4-6 years	1-3 years	Secondary Care								Elderly Care																			Don't know				
40	4-6 years	4-6 years	Secondary Care								Elderly Care					Neurology		Euroqol EQ-5D					Hospital Anxiety and Depression scale (HAD)									I read the questions out t	I helped pa	The partic
41	10 years o	1-3 years	Secondary Care						Cardiovascular									Euroqol EQ-5D														I read the questions out to the partic		The partic
42	10 years o	1-3 years	Secondary Care			Orthopaedics		Rheumatology								Neurology		Euroqol EQ	Health Assessment Questionnaire (HAQ)													I read the questions out t	I helped participants	
43	4-6 years	1-3 years	Secondary Care			Orthopaedics		Rheumatology										Euroqol EQ	Health Assessment Questionnaire (HAQ)						EORTC QLQ - C30 (Core Questionnaire)							I read the questions out t	I helped pa	The partic
44	10 years o	1-3 years	Secondary Care							Oncology								Euroqol EQ-5D																
45	10 years o	10 years or more	Secondary Care							Oncology								Euroqol EQ-5D					Hospital Anxiety and D		EORTC QLQ - C30 (Core Questionnaire)								I helped participants	
46	10 years o	10 years or more	Secondary Care							Oncology								Euroqol EQ-5D					Hospital Anxiety and D		EORTC QLQ - C30 (Core Questionnaire)									
47	10 years o	1-3 years	Secondary Care						Cardiovascular																									
48	10 years o	10 years or more	Secondary Care														Gastroente	Euroqol EQ-5D				SF-36® Health Survey <u>or</u> SF-36v2™ Health Survey												
49	10 years o	4-6 years	Primary Care												Paediatrics			Euroqol EQ-5D																
50	10 years o	4-6 years	Secondary Care			Orthopaed	General M	Rheumatology								Neurology		Euroqol EQ	Health Assessment Questionnaire			SF-36® Health Survey <u>or</u> SF-36v2™ Health Survey												
51	10 years o	1-3 years	Primary Care		General Practice																									Don't know				
52	10 years o	1-3 years	Secondary Care									Respiratory					critical car	Euroqol EQ-5D														I read the questions out t	I helped pa	The partic
53	10 years o	Less than 1 year	Primary Care												Paediatrics	Neurology			Health Assessment Questionnaire (HAQ)													I read the questions out t	I helped pa	The partic
54	10 years o	10 years or more	Secondary Care					Rheumatology									GI	Euroqol EQ	Health Assessment Questionnaire			SF-36® Hea	Hospital Anxiety and Depression scale (HAD)									I read the questions out t	I helped pa	The partic
55	10 years o	Less than 1 year	Secondary Care														gastroenterology														Can't remember			

[illegible]

ID	Thinking about the <u>last</u> clinical trial you worked on that used a quality of life or other patent-reported outcome measure. How would you describe your role?	How long have you worked as a [Q1], in total?	Thinking about the <u>last</u> trial you worked on that used a Quality of Life or other Patient-Reported Outcome measure. Was the trial based in primary care or secondary care?	Thinking about the <u>last</u> trial you worked on that used a Quality of Life or other Patient-Reported Outcome measure. Was the trial based in primary care or secondary care? - Other (please specify)	Which of the following clinical areas did the trial cover? PLEASE TICK ALL THAT APPLY - General Practice	Which of the following clinical areas did the trial cover? PLEASE TICK ALL THAT APPLY - Orthopaedics	Which of the following clinical areas did the trial cover? PLEASE TICK ALL THAT APPLY - General Medicine	Which of the following clinical areas did the trial cover? PLEASE TICK ALL THAT APPLY - Rheumatology	Which of the following clinical areas did the trial cover? PLEASE TICK ALL THAT APPLY - Cardiovascular	Which of the following clinical areas did the trial cover? PLEASE TICK ALL THAT APPLY - Oncology	Which of the following clinical areas did the trial cover? PLEASE TICK ALL THAT APPLY - Elderly Care	Which of the following clinical areas did the trial cover? PLEASE TICK ALL THAT APPLY - Respiratory	Which of the following clinical areas did the trial cover? PLEASE TICK ALL THAT APPLY - Opthalmology	Which of the following clinical areas did the trial cover? PLEASE TICK ALL THAT APPLY - Obstetrics & Gynaecology	Which of the following clinical areas did the trial cover? PLEASE TICK ALL THAT APPLY - Paediatrics	Which of the following clinical areas did the trial cover? PLEASE TICK ALL THAT APPLY - Neurology	Which of the following clinical areas did the trial cover? PLEASE TICK ALL THAT APPLY - DON'T KNOW
1	Data coordinator	1-3 years	Secondary Care							Oncology							
2	Data inputter	1-3 years	Secondary Care							Oncology		Respiratory					
3	Data coordinator	1-3 years	Secondary Care									Respiratory	Opthalmology				
4	Data coordinator	1-3 years	Secondary Care							Oncology							
5	Data coordinator	4-6 years	Secondary Care							Oncology							
6	Data manager	1-3 years	Secondary Care												Paediatrics		
7	Data manager	1-3 years	Primary Care							Oncology							
8	Data manager	7-9 years	Primary Care						Cardiovascular			Respiratory					
9	Data manager	10 years or more	Secondary Care														
10	Data coordinator	4-6 years	Primary Care							Oncology							
11	Data coordinator	4-6 years	Secondary Care							Oncology							
12	Data coordinator	1-3 years	Primary Care							Oncology							
13	Data coordinator																
14	Data manager	10 years or more	Secondary Care			Orthopaedics											
15	Data inputter	10 years or more	Primary Care														DON'T KNOW
16	Data manager	7-9 years	Secondary Care														
17	Data coordinator	4-6 years	Primary Care							Oncology							
18	Data coordinator	10 years or more	Secondary Care				General Medicine										
19	Data coordinator	Less than 1 year	Secondary Care														
20	Data manager	7-9 years	Primary Care								Elderly Care					Neurology	
21	Data coordinator	7-9 years	Secondary Care							Oncology							
22	Data manager	1-3 years	Secondary Care							Oncology							
23	Data manager	Less than 1 year	Primary Care		General Practice							Respiratory					
24	Data manager	10 years or more	Secondary Care												Paediatrics		
25	Data manager	Less than 1 year	Secondary Care														
26	Data inputter	1-3 years	Secondary Care									Respiratory					
27	Data manager	4-6 years	Secondary Care														
28	Data manager	1-3 years	Primary Care		General Practice												
29	Data inputter																
30	Data manager	10 years or more	Primary Care														
31	Data manager	10 years or more	Primary Care							Oncology							
32	Data coordinator	Less than 1 year	Secondary Care							Oncology							
33	Data inputter	4-6 years	Primary Care														DON'T KNOW
34	Data manager	10 years or more	Secondary Care							Oncology				Obstetrics & Gynaecology			
35	Data manager	10 years or more	Primary Care							Oncology							
36	Data manager	10 years or more	Secondary Care											Obstetrics & Gynaecology			
37	Data manager	1-3 years	Secondary Care				General Medicine		Cardiovascular					Obstetrics & Gynaecology			
38	Data coordinator	1-3 years	Primary Care		General Practice												
39	Data inputter	1-3 years	Primary Care					Rheumatology	Cardiovascular								
40	Data manager	4-6 years	Secondary Care								Elderly Care						
41	Data manager																

ID	Which of the following Patient-Reported Outcome Measures did the trial use? PLEASE TICK ALL THAT APPLY - Euroqol EQ-5D	Which of the following Patient-Reported Outcome Measures did the trial use? PLEASE TICK ALL THAT APPLY - Health Assessment Questionnaire (HAQ)	Which of the following Patient-Reported Outcome Measures did the trial use? PLEASE TICK ALL THAT APPLY - Nottingham Health Profile (NHP)	Which of the following Patient-Reported Outcome Measures did the trial use? PLEASE TICK ALL THAT APPLY - SF-12® Health Survey <u>or</u> SF-12v2™ Health Survey	Which of the following Patient-Reported Outcome Measures did the trial use? PLEASE TICK ALL THAT APPLY - SF-36® Health Survey <u>or</u> SF-36v2™ Health Survey	Which of the following Patient-Reported Outcome Measures did the trial use? PLEASE TICK ALL THAT APPLY - Hospital Anxiety and Depression scale (HAD)	Which of the following Patient-Reported Outcome Measures did the trial use? PLEASE TICK ALL THAT APPLY - Arthritis Impact Measurement Scales (AIMS2)	Which of the following Patient-Reported Outcome Measures did the trial use? PLEASE TICK ALL THAT APPLY - EORTC QLQ - C30 (Core Questionnaire)	Which of the following Patient-Reported Outcome Measures did the trial use? PLEASE TICK ALL THAT APPLY - Minnesota Living with Heart Failure © Questionnaire (MLHF)	Which of the following Patient-Reported Outcome Measures did the trial use? PLEASE TICK ALL THAT APPLY - Oxford Hip Score (OHS)	Which of the following Patient-Reported Outcome Measures did the trial use? PLEASE TICK ALL THAT APPLY - Oxford Knee Score (OKS)	Which of the following Patient-Reported Outcome Measures did the trial use? PLEASE TICK ALL THAT APPLY - Roland-Morris Disability Questionnaire (RMDQ)	Which of the following Patient-Reported Outcome Measures did the trial use? PLEASE TICK ALL THAT APPLY - Don't know	Which of the following Patient-Reported Outcome Measures did the trial use? PLEASE TICK ALL THAT APPLY - Can't remember
1														Can't remember
2					SF-36® Health Survey <u>or</u> SF-36v2™ Health Survey			EORTC QLQ - C30 (Core Questionnaire)						
3					SF-36® Health Survey <u>or</u> SF-36v2™ Health Survey									
4	Euroqol EQ-5D							EORTC QLQ - C30 (Core Questionnaire)						
5	Euroqol EQ-5D							EORTC QLQ - C30 (Core Questionnaire)						
6	Euroqol EQ-5D													
7														
8	Euroqol EQ-5D				SF-36® Health Survey <u>or</u> SF-36v2™ Health Survey									
9	Euroqol EQ-5D			SF-12® Health Survey <u>or</u> SF-12v2™ Health Survey										
10	Euroqol EQ-5D													
11								EORTC QLQ - C30 (Core Questionnaire)						
12	Euroqol EQ-5D							EORTC QLQ - C30 (Core Questionnaire)						
13														
14	Euroqol EQ-5D			SF-12® Health Survey <u>or</u> SF-12v2™ Health Survey							Oxford Knee Score (OKS)			
15													Don't know	
16	Euroqol EQ-5D													
17								EORTC QLQ - C30 (Core Questionnaire)						
18	Euroqol EQ-5D													
19								EORTC QLQ - C30 (Core Questionnaire)						
20	Euroqol EQ-5D													
21								EORTC QLQ - C30 (Core Questionnaire)						
22	Euroqol EQ-5D													
23	Euroqol EQ-5D													
24		Health Assessment Questionnaire (HAQ)												
25	Euroqol EQ-5D					Hospital Anxiety and Depression scale (HAD)								
26					SF-36® Health Survey <u>or</u> SF-	Hospital Anxiety and Depression scale (HAD)								
27	Euroqol EQ-5D			SF-12® Health Survey <u>or</u> SF-12v2™ Health Survey										
28	Euroqol EQ-5D													
29														
30	Euroqol EQ-5D													
31	Euroqol EQ-5D					Hospital Anxiety and Depression scale (HAD)		EORTC QLQ - C30 (Core Questionnaire)						
32														Can't remember
33	Euroqol EQ-5D													
34	Euroqol EQ-5D													
35	Euroqol EQ-5D			SF-12® Health Survey <u>or</u> SF-12v2™ Health Survey		Hospital Anxiety and Depression scale (HAD)								
36	Euroqol EQ-5D			SF-12® Health Survey <u>or</u> SF-12v2™ Health Survey										
37	Euroqol EQ-5D													
38	Euroqol EQ-5D													
39				SF-12® Health Survey <u>or</u> SF-	SF-36® Health Survey <u>or</u> SF-36v2™ Health Survey									
40	Euroqol EQ-5D													
41														

ID	Thinking about the <u>last</u> trial you worked on that used a Quality of Life or other Patient-Reported Outcome measure. When the Quality of Life/Patient-Reported Outcome questionnaire data were <u>inputted</u>, which of the following occurred? PLEASE TICK ALL THAT APPLY - The questionnaire was checked to see if the participant had completed all questions.	Thinking about the <u>last</u> trial you worked on that used a Quality of Life or other Patient-Reported Outcome measure. When the Quality of Life/Patient-Reported Outcome questionnaire data were <u>inputted</u>, which of the following occurred? PLEASE TICK ALL THAT APPLY - If items were found to be missing, trial participants were followed up in some way (e.g. by post, by phone or via their research nurse) in order to complete the questionnaire.	Thinking about the <u>last</u> trial you worked on that used a Quality of Life or other Patient-Reported Outcome measure. When the Quality of Life/Patient-Reported Outcome questionnaire data were <u>inputted</u>, which of the following occurred? PLEASE TICK ALL THAT APPLY - The questionnaire was checked for scoring errors (e.g. two answers given instead of one, or reversed scoring).	Thinking about the <u>last</u> trial you worked on that used a Quality of Life or other Patient-Reported Outcome measure. When the Quality of Life/Patient-Reported Outcome questionnaire data were <u>inputted</u>, which of the following occurred? PLEASE TICK ALL THAT APPLY - If scoring errors were detected, trial participants were followed up in some way (e.g. by post, by phone or via their research nurse) in order to correct them.	Thinking about the <u>last</u> trial you worked on that used a Quality of Life or other Patient-Reported Outcome measure. When the Quality of Life/Patient-Reported Outcome questionnaire data were <u>inputted</u>, which of the following occurred? PLEASE TICK ALL THAT APPLY - Other (please specify)	Again, thinking about the <u>last</u> trial you worked on that used a Quality of Life or other Patient-Reported Outcome measure. Please read the following statements. In each case, please answer 'yes', 'no', 'don't know', or 'not applicable'. - The trial **protocol** included information about Quality of Life/Patient-Reported Outcome data inputting.	Again, thinking about the <u>last</u> trial you worked on that used a Quality of Life or other Patient-Reported Outcome measure. Please read the following statements. In each case, please answer 'yes', 'no', 'don't know', or 'not applicable'. - I felt the trial **protocol** content covering Quality of Life/Patient-Reported Outcome data inputting was adequate.	Again, thinking about the <u>last</u> trial you worked on that used a Quality of Life or other Patient-Reported Outcome measure. Please read the following statements. In each case, please answer 'yes', 'no', 'don't know', or 'not applicable'. - I received **trial training** which included information on Quality of Life/Patient-Reported Outcome data inputting.	Again, thinking about the <u>last</u> trial you worked on that used a Quality of Life or other Patient-Reported Outcome measure. Please read the following statements. In each case, please answer 'yes', 'no', 'don't know', or 'not applicable'. - I felt the **trial training** I received covering Quality of Life/Patient-Reported Outcome data inputting was adequate.	Again, thinking about the <u>last</u> trial you worked on that used a Quality of Life or other Patient-Reported Outcome measure. Please read the following statements. In each case, please answer 'yes', 'no', 'don't know', or 'not applicable'. - I had access to a **standard operating procedure** which included information on Quality of Life/Patient-Reported Outcome data inputting.	Again, thinking about the <u>last</u> trial you worked on that used a Quality of Life or other Patient-Reported Outcome measure. Please read the following statements. In each case, please answer 'yes', 'no', 'don't know', or 'not applicable'. - I felt the **standard operating procedure** covering Quality of Life/Patient-Reported Outcome data inputting was adequate.
1	The questionnaire was checked to see	If items were found to be missing, trial participants were followed up in some way (e.g. by post, by phone or via their research nurse) in order to complete t				Yes	Yes	Yes	Yes	Yes	Yes
2	The questionnaire was checked to see if the participant had completed all qu		The questionnaire was checked for scoring errors (e.g. two answers given instead of one, or reversed scoring).			No	Not applicable	No	Not applicable	Yes	Yes
3	The questionnaire was checked to see	If items were found to be missing, trial	The questionnaire was checked for sc	If scoring errors were detected, trial participants were followed up in some wa		No	Not applicable	No	Not applicable	No	Yes
4											
5					Data has not been inputted yet - but a	No	Not applicable	No	Not applicable	No	Not applicable
6	The questionnaire was checked to see if the participant had completed all questions.					No	Not applicable	No	Not applicable	Yes	Yes
7											
8	The questionnaire was checked to see	If items were found to be missing, trial participants were followed up in some way (e.g. by post, by phone or via their research nurse) in order to complete t				Yes	Yes	Not applicable	Not applicable	Yes	Yes
9					Data entry conventions applied, missi	No	Yes	Yes		Yes	Yes
10	The questionnaire was checked to see if the participant had completed all qu		The questionnaire was checked for scoring errors (e.g. two answers given instead of one, or reversed scoring).			No	Not applicable	No	Not applicable	No	Not applicable
11	The questionnaire was checked to see if the participant had completed all qu		The questionnaire was checked for scoring errors (e.g. two answers given in		If scoring errors noted ie 2 the worst s	Not applicable	Not applicable	Yes	Yes	No	Not applicable
12	The questionnaire was checked to see if the participant had completed all qu		The questionnaire was checked for scoring errors (e.g. two answers given instead of one, or reversed scoring).			Yes	Yes	Yes	Yes	Yes	Yes
13											
14											
15			The questionnaire was checked for scoring errors (e.g. two answers given instead of one, or reversed scoring).			Don't Know	Don't Know	Yes	Yes	Yes	Don't Know
16			The questionnaire was checked for scoring errors (e.g. two answers given in		Data was entered as seen. We don't c	Yes	Yes	Not applicable	Not applicable	Yes	Yes
17	The questionnaire was checked to see if the participant had completed all qu		The questionnaire was checked for scoring errors (e.g. two answers given instead of one, or reversed scoring).			Don't Know	Yes	No	Yes	No	Not applicable
18	The questionnaire was checked to see	If items were found to be missing, trial	The questionnaire was checked for sc	If scoring errors were detected, trial participants were followed up in some wa		Yes	No	No	Not applicable	No	Not applicable
19											
20	The questionnaire was checked to see	If items were found to be missing, trial	The questionnaire was checked for sc	If scoring errors were detected, trial participants were followed up in some wa		No	No	Not applicable	Not applicable	Yes	Yes
21					entered as received	Yes	Yes	Yes	Yes	No	Yes
22	The questionnaire was checked to see if the participant had completed all qu		The questionnaire was checked for scoring errors (e.g. two answers given instead of one, or reversed scoring).			No	No	No	Not applicable	Yes	Yes
23	The questionnaire was checked to see if the participant had completed all questions.					Yes	Yes	Not applicable	Not applicable	Not applicable	Not applicable
24											
25											
26			The questionnaire was checked for scoring errors (e.g. two answers given instead of one, or reversed scoring).			No	Not applicable	No		No	Not applicable
27											
28	The questionnaire was checked to see if the participant had completed all questions.					Yes	Yes	No	Not applicable	Not applicable	Not applicable
29											
30			The questionnaire was checked for scoring errors (e.g. two answers given instead of one, or reversed scoring).			Yes	Yes	Yes	Yes	Yes	Yes
31	The questionnaire was checked to see if the participant had completed all qu		The questionnaire was checked for scoring errors (e.g. two answers given instead of one, or reversed scoring).			No	Not applicable	No	Not applicable	Yes	Yes
32											
33						Don't Know	Don't Know	Yes	Yes	Don't Know	Don't Know
34						No	Not applicable	Not applicable	Not applicable	No	Not applicable
35		If items were found to be missing, trial	The questionnaire was checked for scoring errors (e.g. two answers given instead of one, or reversed scoring).			Yes	Yes	No	No	No	No
36	The questionnaire was checked to see	If items were found to be missing, trial	The questionnaire was checked for sc	If scoring errors were detected, trial participants were followed up in some wa		No	No	No	No	No	No
37			The questionnaire was checked for scoring errors (e.g. two answers given instead of one, or reversed scoring).			Yes	Yes	Yes	Yes	Yes	Yes
38	The questionnaire was checked to see if the participant had completed all qu		The questionnaire was checked for scoring errors (e.g. two answers given instead of one, or reversed scoring).								
39			The questionnaire was checked for scoring errors (e.g. two answers given instead of one, or reversed scoring).			Yes	Don't Know	No	Not applicable	No	Don't Know
40	The questionnaire was checked to see if the participant had completed all questions.					Yes	No	Not applicable	Not applicable	No	Not applicable
41											

ID	Some data managers/inputters we have spoken to have reported encountering Quality of Life/Patient-Reported Outcome data which raised concern for the wellbeing of the trial participant in some way. This information has been termed: ‘concerning’ Patient-Reported Outcome information. In these reports, ‘concerning’ Patient-Reported Outcome information may have been extreme questionnaire scores, or sometimes a participant might have written additional information on the questionnaire which raised concern (or attached a letter). Thinking about your <u>general</u> experience. Have you ever encountered any ‘concerning’ Patient-Reported Outcome information within a trial?	Have you ever taken action in response to ‘concerning’ Patient-Reported Outcome information you have encountered within a trial, in order to assist a trial participant?	Were you able to record <u>all</u> action(s) taken in response to viewing ‘concerning’ Patient-Reported Outcome information, in the trial documentation?	If you were to encounter ‘concerning’ Patient-Reported Outcome information <u>in a future trial</u>, for example, evidence of anxiety or depression, which of the following might you consider doing? PLEASE TICK ALL THAT APPLY - I would not intervene, there is nothing I could do.	If you were to encounter ‘concerning’ Patient-Reported Outcome information <u>in a future trial</u>, for example, evidence of anxiety or depression, which of the following might you consider doing? PLEASE TICK ALL THAT APPLY - I would not intervene, it is the responsibility of the trial participant's GP and regular healthcare team to monitor and deal with quality of life related disorders such as anxiety and depression, not the trial staff.	If you were to encounter ‘concerning’ Patient-Reported Outcome information <u>in a future trial</u>, for example, evidence of anxiety or depression, which of the following might you consider doing? PLEASE TICK ALL THAT APPLY - I would discuss the findings with my line manager in the trial, or with the PI.	If you were to encounter ‘concerning’ Patient-Reported Outcome information <u>in a future trial</u>, for example, evidence of anxiety or depression, which of the following might you consider doing? PLEASE TICK ALL THAT APPLY - I would discuss the findings with the participants research nurse.	If you were to encounter ‘concerning’ Patient-Reported Outcome information <u>in a future trial</u>, for example, evidence of anxiety or depression, which of the following might you consider doing? PLEASE TICK ALL THAT APPLY - I would discuss the findings with a data manager/inputter colleague.
1	Yes	No	No	I would not intervene, there is nothing I could do.			I would discuss the findings with the participants research nurse.	
2	No					I would discuss the findings with my li	I would discuss the findings with the participants research nurse.	
3	Yes	Yes	No			I would discuss the findings with my li	I would discuss the findings with the participants research nurse.	
4								
5	No			I would not intervene, there is nothing I could do.				
6	No				I would not intervene, it is the responsibility of the trial participant's GP and regular healthcare team to monitor and deal with quality of life related disorders			
7								
8	No							
9	No				I would not intervene, it is the responsibility of the trial participant's GP and regular healthcare team to monitor and deal with quality of life related disorders			
10	No				I would not intervene, it is the responsibility of the trial participant's GP and regular healthcare team to monitor and deal with quality of life related disorders			
11	No					I would discuss the findings with my line manager in the trial, or with the PI.		
12	Yes	No	Not applicable		I would not intervene, it is the responsibility of the trial participant's GP and regular healthcare team to monitor and deal with quality of life related disorders			
13								
14								
15	Don't know					I would discuss the findings with my line manager in the trial, or with the PI.		
16	Yes	Yes	Yes					
17	Yes	No	Not applicable			I would discuss the findings with my li	I would discuss the findings with the participants research nurse.	
18	No					I would discuss the findings with my li	I would discuss the findings with the participants research nurse.	
19								
20	Yes	Yes	Yes			I would discuss the findings with my li	I would discuss the findings with the participants research nurse.	
21	Don't know				I would not intervene, it is the responsibility of the trial participant's GP and regular healthcare team to monitor and deal with quality of life related disorders			
22	Yes	No	No			I would discuss the findings with my li	I would discuss the findings with the p	I would discuss the findings with a dat
23	No				I would not intervene, it is the responsibility of the trial participant's GP and regular healthcare team to monitor and deal with quality of life related disorders			
24								
25								
26	Yes	Yes	Yes			I would discuss the findings with my li	I would discuss the findings with the participants research nurse.	
27								
28	No					I would discuss the findings with my li	I would discuss the findings with the participants research nurse.	
29								
30	No					I would discuss the findings with my line manager in the trial, or with the PI.		
31	Yes	Yes	Not applicable			I would discuss the findings with my line manager in the trial, or with the PI.		
32								
33	Yes	No	Not applicable		I would not intervene, it is the respons	I would discuss the findings with my line manager in the trial, or with the PI.		
34	No				I would not intervene, it is the responsibility of the trial participant's GP and regular healthcare team to monitor and deal with quality of life related disorders			
35	Yes	Yes	Yes			I would discuss the findings with my li	I would discuss the findings with the participants research nurse.	
36	Yes	Yes	No					
37	Yes	No	Not applicable		I would not intervene, it is the responsibility of the trial participant's GP and re		I would discuss the findings with the participants research nurse.	
38	Yes							
39	No				I would not intervene, it is the responsibility of the trial participant's GP and regular healthcare team to monitor and deal with quality of life related disorders			
40	No				I would not intervene, it is the responsibility of the trial participant's GP and regular healthcare team to monitor and deal with quality of life related disorders			
41								

ID	Please read the following statements. In each case, please answer 'yes', 'no', or 'don't know'. - There is usually <u>specific guidance</u> on dealing with **'concerning'** Patient-Reported Outcome information contained in trial protocols.	Please read the following statements. In each case, please answer 'yes', 'no', or 'don't know'. - I usually receive <u>training</u> on what to do if I encounter **'concerning'** Patient-Reported Outcome information.	Please read the following statements. In each case, please answer 'yes', 'no', or 'don't know'. - I feel <u>confident</u> about dealing with **'concerning'** Patient-Reported Outcome trial information.	Thinking about the <u>future</u>. Please read the following statements. In each case, please indicate whether you 'strongly agree', 'agree', have 'no opinion', 'disagree' or 'strongly disagree' with the statement. - There should be more **protocol content and trial training** for data managers/inputters, covering Quality of Life/Patient-Reported Outcome measurement.	Thinking about the <u>future</u>. Please read the following statements. In each case, please indicate whether you 'strongly agree', 'agree', have 'no opinion', 'disagree' or 'strongly disagree' with the statement. - There should be **site manuals or standard operating procedures** available to data mangers/inputters that include information on Quality of Life/Patient-Reported Outcome administration in the trial.	Thinking about the <u>future</u>. Please read the following statements. In each case, please indicate whether you 'strongly agree', 'agree', have 'no opinion', 'disagree' or 'strongly disagree' with the statement. - There should be <u>specific</u> protocol content and trial training for data managers/inputters on how to deal with **'concerning' Patient-Reported Outcome information** in trials.
1	Yes	Yes	Yes	No Opinion	No Opinion	Agree
2	Yes	No	Don't know	Agree	Strongly Agree	Strongly Agree
3	No	No	Don't know	Agree	Agree	Strongly Agree
4						
5	No	No	No	Agree	Strongly Agree	Disagree
6	No	No	Don't know	Agree	Agree	Disagree
7						
8	Don't know	Don't know	Don't know	Strongly Agree	Strongly Agree	Strongly Agree
9	No	No	No	Agree	Agree	Disagree
10	No	No	Don't know	No Opinion	No Opinion	No Opinion
11	Yes	Yes	Yes	Agree	Agree	Agree
12	Don't know	No	No	Agree	Agree	Agree
13						
14						
15	Yes	Yes	Yes	Agree	Agree	Strongly Agree
16	Yes	Yes	Yes	Disagree	Agree	Agree
17	Don't know	No	Don't know	Strongly Agree	Strongly Agree	Strongly Agree
18	No	No	Yes	Agree	Agree	Agree
19						
20	No	No	Yes	Agree	Agree	No Opinion
21	No	No	Don't know	Agree	Agree	Agree
22	No	No	No	Disagree	Strongly Agree	Strongly Disagree
23	No	No	No	Agree	Agree	No Opinion
24						
25						
26	Yes	Yes	Yes	Disagree	Disagree	Disagree
27						
28	Don't know	No	No	Agree	Agree	Agree
29						
30	Yes	Yes	Yes	Agree	Strongly Agree	Strongly Agree
31	No	No	Yes	Disagree	Agree	Agree
32						
33	Don't know	No	Don't know	Strongly Agree	Agree	Agree
34	No	No	Don't know	Disagree	Agree	No Opinion
35	No	No	Yes	Agree	Agree	Strongly Agree
36						
37	Yes	No	Yes	Disagree	Agree	Agree
38						
39	Don't know	No	No	Agree	Agree	Agree
40	No	No	No	Agree	No Opinion	No Opinion
41						

ID	Thinking about the <u>future</u>. In your opinion, what particular Quality of Life/Patient-Reported Outcome data collection guidance do you feel is <u>essential</u> in helping you do your job well, and should be included the <u>trial protocol</u>, what should be included in <u>trial training</u>, and what should be included in a <u>standard operating procedure</u>? PLEASE TICK ALL THAT APPLY - How to input Quality of Life/Patient-Reported Outcome data into the database. - Trial Protocol	Thinking about the <u>future</u>. In your opinion, what particular Quality of Life/Patient-Reported Outcome data collection guidance do you feel is <u>essential</u> in helping you do your job well, and should be included the <u>trial protocol</u>, what should be included in <u>trial training</u>, and what should be included in a <u>standard operating procedure</u>? PLEASE TICK ALL THAT APPLY - How to input Quality of Life/Patient-Reported Outcome data into the database. - Trial Training	Thinking about the <u>future</u>. In your opinion, what particular Quality of Life/Patient-Reported Outcome data collection guidance do you feel is <u>essential</u> in helping you do your job well, and should be included the <u>trial protocol</u>, what should be included in <u>trial training</u>, and what should be included in a <u>standard operating procedure</u>? PLEASE TICK ALL THAT APPLY - How to input Quality of Life/Patient-Reported Outcome data into the database. - Standard Operating Procedure	Thinking about the <u>future</u>. In your opinion, what particular Quality of Life/Patient-Reported Outcome data collection guidance do you feel is <u>essential</u> in helping you do your job well, and should be included the <u>trial protocol</u>, what should be included in <u>trial training</u>, and what should be included in a <u>standard operating procedure</u>? PLEASE TICK ALL THAT APPLY - What to do if there is missing data. - Trial Protocol	Thinking about the <u>future</u>. In your opinion, what particular Quality of Life/Patient-Reported Outcome data collection guidance do you feel is <u>essential</u> in helping you do your job well, and should be included the <u>trial protocol</u>, what should be included in <u>trial training</u>, and what should be included in a <u>standard operating procedure</u>? PLEASE TICK ALL THAT APPLY - What to do if there is missing data. - Trial Training	Thinking about the <u>future</u>. In your opinion, what particular Quality of Life/Patient-Reported Outcome data collection guidance do you feel is <u>essential</u> in helping you do your job well, and should be included the <u>trial protocol</u>, what should be included in <u>trial training</u>, and what should be included in a <u>standard operating procedure</u>? PLEASE TICK ALL THAT APPLY - What to do if there is missing data. - Standard Operating Procedure
1		Trial Training	Standard Operating Procedure		Trial Training	Standard Operating Procedure
2			Standard Operating Procedure			Standard Operating Procedure
3			Standard Operating Procedure			Standard Operating Procedure
4						
5		Trial Training				Standard Operating Procedure
6		Trial Training	Standard Operating Procedure		Trial Training	Standard Operating Procedure
7						
8		Trial Training	Standard Operating Procedure		Trial Training	Standard Operating Procedure
9			Standard Operating Procedure			Standard Operating Procedure
10		Trial Training			Trial Training	
11		Trial Training	Standard Operating Procedure		Trial Training	Standard Operating Procedure
12		Trial Training			Trial Training	
13						
14						
15			Standard Operating Procedure			Standard Operating Procedure
16		Trial Training	Standard Operating Procedure		Trial Training	Standard Operating Procedure
17		Trial Training			Trial Training	
18		Trial Training		Trial Protocol	Trial Training	
19						
20	Trial Protocol		Standard Operating Procedure			Standard Operating Procedure
21		Trial Training			Trial Training	
22		Trial Training	Standard Operating Procedure			Standard Operating Procedure
23	Trial Protocol	Trial Training	Standard Operating Procedure		Trial Training	Standard Operating Procedure
24						
25						
26						Standard Operating Procedure
27						
28		Trial Training	Standard Operating Procedure		Trial Training	Standard Operating Procedure
29						
30		Trial Training	Standard Operating Procedure		Trial Training	Standard Operating Procedure
31		Trial Training	Standard Operating Procedure		Trial Training	Standard Operating Procedure
32						
33		Trial Training	Standard Operating Procedure		Trial Training	Standard Operating Procedure
34		Trial Training			Trial Training	Standard Operating Procedure
35		Trial Training	Standard Operating Procedure		Trial Training	Standard Operating Procedure
36						
37		Trial Training			Trial Training	Standard Operating Procedure
38						
39		Trial Training				Standard Operating Procedure
40	Trial Protocol	Trial Training	Standard Operating Procedure	Trial Protocol	Trial Training	Standard Operating Procedure
41						

ID	Thinking about the <u>future</u>. In your opinion, what particular Quality of Life/Patient-Reported Outcome data collection guidance do you feel is <u>essential</u> in helping you do your job well, and should be included the <u>trial protocol</u>, what should be included in <u>trial training</u>, and what should be included in a <u>standard operating procedure</u>? PLEASE TICK ALL THAT APPLY - What to do in the event of scoring errors (e.g. two answers instead of one, or reversed scoring). - Trial Protocol	Thinking about the <u>future</u>. In your opinion, what particular Quality of Life/Patient-Reported Outcome data collection guidance do you feel is <u>essential</u> in helping you do your job well, and should be included the <u>trial protocol</u>, what should be included in <u>trial training</u>, and what should be included in a <u>standard operating procedure</u>? PLEASE TICK ALL THAT APPLY - What to do in the event of scoring errors (e.g. two answers instead of one, or reversed scoring). - Trial Training	Thinking about the <u>future</u>. In your opinion, what particular Quality of Life/Patient-Reported Outcome data collection guidance do you feel is <u>essential</u> in helping you do your job well, and should be included the <u>trial protocol</u>, what should be included in <u>trial training</u>, and what should be included in a <u>standard operating procedure</u>? PLEASE TICK ALL THAT APPLY - What to do in the event of scoring errors (e.g. two answers instead of one, or reversed scoring). - Standard Operating Procedure	Thinking about the <u>future</u>. In your opinion, what particular Quality of Life/Patient-Reported Outcome data collection guidance do you feel is <u>essential</u> in helping you do your job well, and should be included the <u>trial protocol</u>, what should be included in <u>trial training</u>, and what should be included in a <u>standard operating procedure</u>? PLEASE TICK ALL THAT APPLY - What to do if participants write additional information on their questionnaires (or attach a letter). - Trial Protocol	Thinking about the <u>future</u>. In your opinion, what particular Quality of Life/Patient-Reported Outcome data collection guidance do you feel is <u>essential</u> in helping you do your job well, and should be included the <u>trial protocol</u>, what should be included in <u>trial training</u>, and what should be included in a <u>standard operating procedure</u>? PLEASE TICK ALL THAT APPLY - What to do if participants write additional information on their questionnaires (or attach a letter). - Trial Training	Thinking about the <u>future</u>. In your opinion, what particular Quality of Life/Patient-Reported Outcome data collection guidance do you feel is <u>essential</u> in helping you do your job well, and should be included the <u>trial protocol</u>, what should be included in <u>trial training</u>, and what should be included in a <u>standard operating procedure</u>? PLEASE TICK ALL THAT APPLY - What to do if participants write additional information on their questionnaires (or attach a letter). - Standard Operating Procedure
1		Trial Training	Standard Operating Procedure		Trial Training	Standard Operating Procedure
2			Standard Operating Procedure			Standard Operating Procedure
3			Standard Operating Procedure		Trial Training	
4						
5			Standard Operating Procedure			Standard Operating Procedure
6		Trial Training	Standard Operating Procedure		Trial Training	
7						
8		Trial Training	Standard Operating Procedure		Trial Training	Standard Operating Procedure
9			Standard Operating Procedure			Standard Operating Procedure
10		Trial Training			Trial Training	
11		Trial Training	Standard Operating Procedure		Trial Training	Standard Operating Procedure
12		Trial Training			Trial Training	
13						
14						
15			Standard Operating Procedure			Standard Operating Procedure
16		Trial Training			Trial Training	
17		Trial Training			Trial Training	
18	Trial Protocol	Trial Training		Trial Protocol	Trial Training	
19						
20			Standard Operating Procedure			Standard Operating Procedure
21		Trial Training		Trial Protocol	Trial Training	
22			Standard Operating Procedure		Trial Training	Standard Operating Procedure
23		Trial Training	Standard Operating Procedure		Trial Training	Standard Operating Procedure
24						
25						
26						Standard Operating Procedure
27						
28		Trial Training	Standard Operating Procedure		Trial Training	Standard Operating Procedure
29						
30		Trial Training	Standard Operating Procedure	Trial Protocol	Trial Training	Standard Operating Procedure
31			Standard Operating Procedure			Standard Operating Procedure
32						
33		Trial Training	Standard Operating Procedure		Trial Training	Standard Operating Procedure
34		Trial Training	Standard Operating Procedure		Trial Training	Standard Operating Procedure
35		Trial Training	Standard Operating Procedure		Trial Training	Standard Operating Procedure
36						
37		Trial Training			Trial Training	
38						
39			Standard Operating Procedure			Standard Operating Procedure
40	Trial Protocol	Trial Training	Standard Operating Procedure		Trial Training	
41						

ID	Thinking about the <u>future</u>. In your opinion, what particular Quality of Life/Patient-Reported Outcome data collection guidance do you feel is <u>essential</u> in helping you do your job well, and should be included the <u>trial protocol</u>, what should be included in <u>trial training</u>, and what should be included in a <u>standard operating procedure</u>? PLEASE TICK ALL THAT APPLY - When action should be taken in the event of extreme questionnaire scores. - Trial Protocol	Thinking about the <u>future</u>. In your opinion, what particular Quality of Life/Patient-Reported Outcome data collection guidance do you feel is <u>essential</u> in helping you do your job well, and should be included the <u>trial protocol</u>, what should be included in <u>trial training</u>, and what should be included in a <u>standard operating procedure</u>? PLEASE TICK ALL THAT APPLY - When action should be taken in the event of extreme questionnaire scores. - Trial Training	Thinking about the <u>future</u>. In your opinion, what particular Quality of Life/Patient-Reported Outcome data collection guidance do you feel is <u>essential</u> in helping you do your job well, and should be included the <u>trial protocol</u>, what should be included in <u>trial training</u>, and what should be included in a <u>standard operating procedure</u>? PLEASE TICK ALL THAT APPLY - When action should be taken in the event of extreme questionnaire scores. - Standard Operating Procedure	Thinking about the <u>future</u>. In your opinion, what particular Quality of Life/Patient-Reported Outcome data collection guidance do you feel is <u>essential</u> in helping you do your job well, and should be included the <u>trial protocol</u>, what should be included in <u>trial training</u>, and what should be included in a <u>standard operating procedure</u>? PLEASE TICK ALL THAT APPLY - Who to speak to if there is concern about a participants Quality of Life/Patient-Reported Outcome data. - Trial Protocol	Thinking about the <u>future</u>. In your opinion, what particular Quality of Life/Patient-Reported Outcome data collection guidance do you feel is <u>essential</u> in helping you do your job well, and should be included the <u>trial protocol</u>, what should be included in <u>trial training</u>, and what should be included in a <u>standard operating procedure</u>? PLEASE TICK ALL THAT APPLY - Who to speak to if there is concern about a participants Quality of Life/Patient-Reported Outcome data. - Trial Training	Thinking about the <u>future</u>. In your opinion, what particular Quality of Life/Patient-Reported Outcome data collection guidance do you feel is <u>essential</u> in helping you do your job well, and should be included the <u>trial protocol</u>, what should be included in <u>trial training</u>, and what should be included in a <u>standard operating procedure</u>? PLEASE TICK ALL THAT APPLY - Who to speak to if there is concern about a participants Quality of Life/Patient-Reported Outcome data. - Standard Operating Procedure
1	Trial Protocol	Trial Training	Standard Operating Procedure	Trial Protocol	Trial Training	Standard Operating Procedure
2	Trial Protocol			Trial Protocol		
3			Standard Operating Procedure	Trial Protocol		
4						
5		Trial Training			Trial Training	
6		Trial Training			Trial Training	
7						
8	Trial Protocol	Trial Training	Standard Operating Procedure		Trial Training	Standard Operating Procedure
9						
10		Trial Training			Trial Training	
11		Trial Training	Standard Operating Procedure	Trial Protocol	Trial Training	Standard Operating Procedure
12		Trial Training			Trial Training	
13						
14						
15			Standard Operating Procedure			Standard Operating Procedure
16	Trial Protocol	Trial Training			Trial Training	
17		Trial Training			Trial Training	
18	Trial Protocol	Trial Training		Trial Protocol	Trial Training	
19						
20			Standard Operating Procedure			Standard Operating Procedure
21	Trial Protocol			Trial Protocol		
22		Trial Training	Standard Operating Procedure		Trial Training	Standard Operating Procedure
23		Trial Training			Trial Training	
24						
25						
26			Standard Operating Procedure			Standard Operating Procedure
27						
28	Trial Protocol	Trial Training	Standard Operating Procedure		Trial Training	Standard Operating Procedure
29						
30		Trial Training	Standard Operating Procedure	Trial Protocol	Trial Training	Standard Operating Procedure
31		Trial Training			Trial Training	Standard Operating Procedure
32						
33		Trial Training	Standard Operating Procedure		Trial Training	Standard Operating Procedure
34		Trial Training	Standard Operating Procedure		Trial Training	Standard Operating Procedure
35	Trial Protocol	Trial Training	Standard Operating Procedure	Trial Protocol	Trial Training	Standard Operating Procedure
36						
37		Trial Training		Trial Protocol	Trial Training	
38						
39	Trial Protocol			Trial Protocol		
40		Trial Training			Trial Training	
41						

National Trial Manager Survey

Q1. First, some questions about yourself. How long in total have you worked as a trial manager?

Answer Options	Response Perce	Response Count
Less than 1 year	9.3 %	12
1-3 years	32.6 %	42
4-6 years	24.0 %	31
7-9 years	9.3 %	12
10 years or more	24.8 %	32
	answered ques	129
	skipped questic	0

Q2. To which of the following age groups do you belong?

Answer Options	Response Perce	Response Count
25 or younger	3.1 %	4
26-35	39.5 %	51
36-45	33.3 %	43
46-55	17.8 %	23
56 or older	6.2 %	8
	answered ques	129
	skipped questic	0

Q3. Thinking about the last trial you worked on that used a Quality of Life or other Patient-Reported Outcome measure. Which of the following clinical areas did the trial cover? PLEASE TICK ALL THAT APPLY

Answer Options	Response Perce	Response Count
General Practice	23.0 %	23
Orthopaedics	7.0 %	7
General Medicine	7.0 %	7
Rheumatology	6.0 %	6
Cardiovascular	10.0 %	10
Oncology	28.0 %	28
Elderly Care	10.0 %	10
Respiratory	8.0 %	8
Opthalmology	4.0 %	4
Obstetrics & Gynaecology	7.0 %	7
Paediatrics	9.0 %	9
Neurology	9.0 %	9
Comments		50
	answered ques	100
	skipped questic	29

Q4. Was the trial based in primary or secondary care?

Answer Options	Response Perce	Response Count
Primary Care	37.9 %	47
Secondary Care	62.1 %	77
Comments		8
	answered ques	124
	skipped questic	5

Q5. Which of the following Patient-Reported Outcome Measures did the trial use? PLEASE TICK ALL THAT APPLY

Answer Options	Response Perce	Response Count
Euroqol EQ-5D	82.5 %	99
Health Assessment Questionnaire (HAQ)	3.3 %	4
Nottingham Health Profile (NHP)	0.0 %	0
SF-12® Health Survey or SF-12v2™ Health Survey	18.3 %	22
SF-36® Health Survey or SF-36v2™ Health Survey	14.2 %	17
Hospital Anxiety and Depression scale (HAD)	17.5 %	21
Arthritis Impact Measurement Scales (AIMS2)	0.0 %	0
EORTC QLQ - C30 (Core Questionnaire)	15.0 %	18
Minnesota Living with Heart Failure © Questionnaire (MLHF)	0.8 %	1
Oxford Hip Score (OHS)	0.0 %	0
Oxford Knee Score (OKS)	0.0 %	0
Roland-Morris Disability Questionnaire (RMDQ)	1.7 %	2
Don't know	2.5 %	3
Can't remember	2.5 %	3
Comments		56
	answered ques	120
	skipped questic	9

Q6. During the trial, were the staff involved in data collection given instructions on how to administer the quality of life/ patient-reported outcome questionnaire?

Answer Options	Response Perce	Response Count
No	29.1 %	37
Yes	70.9 %	90
Comments		32
	answered ques	127
	skipped questic	2

Q7. Again, thinking about the same trial. What particular information on Quality of Life/Patient-Reported Outcome measurement was given to the data collection staff? Please read the options below and in each case select either 'included in trial protocol, training or SOP', or 'not included'.

Answer Options	Included in trial	Not included	Response (	Included ir	Not included %
The purpose and/or Importance of Quality of Life/Patient-Reported Outcome data to the trial.	71	11	82	86.6%	13.4%
Relevance and reasoning behind individual Quality of Life/Patient-Reported Outcome questions.	43	39	82	52.4%	47.6%
When to administer the questionnaire (time points).	84	0	84	100.0%	0.0%
When to administer the questionnaire during the clinic appointment (before/during/after the consultation).	53	25	78	67.9%	32.1%
How much assistance to give the participant during questionnaire completion.	52	30	82	63.4%	36.6%
How to check for, and deal with, missing Quality of Life/Patient-Reported Outcome data.	48	34	82	58.5%	41.5%
How to deal with Quality of Life/Patient-Reported Outcome information that raises concern for the wellbeing of the trial participant (e.g. a questionnaire indicating severe anxiety or depression).	31	50	81	38.3%	61.7%
What to do if participants write additional information on their questionnaires (or attach a letter).	23	59	82	28.0%	72.0%
Comments			12		
		answered que	84		
		skipped questi	45		

Q8. Some research nurses/data mangers we have spoken to have reported encountering Quality of Life/Patient-Reported Outcome questionnaires containing answers which raise concern for the wellbeing of the trial participant in some way. This information has been termed: 'concerning' Patient-Reported Outcome information. In these reports, 'concerning' Patient-Reported Outcome information may simply have been particularly extreme questionnaire scores, or sometimes a participant might have written additional information on the questionnaire which raised concern (or attached a letter); finally, some research nurses reported becoming concerned by things that the trial participant said to them either during, or after, the completion of the questionnaire. Have you ever encountered/been made aware of any 'concerning' Patient-Reported Outcome information within a trial?

Answer Options	Response Perce	Response Count
No	52.1 %	62
Yes	46.2 %	55
Don't know	1.7 %	2
	answered ques	119
	skipped questic	10

Q9. Have you ever taken action in response to 'concerning' Patient-Reported Outcome information you have encountered/ been made aware of within a trial, in order to assist a trial participant?

Answer Options	Response Perce	Response Count
No	14.8 %	8
Yes	85.2 %	46
Comments		37
	answered ques	54
	skipped questic	75

Q10. Was there a mechanism in place to record all action(s) taken in response to the 'concerning' Patient-Reported Outcome information, in the trial documentation?

Answer Options	Response Perce	Response Count
No	50.9 %	27
Yes	47.2 %	25
Not applicable	1.9 %	1
Comments		18
	answered ques	53
	skipped questic	76

Q11. If your data collection staff were to encounter 'concerning' Patient-Reported Outcome information in a future trial, for example, evidence of anxiety or depression, which of the following would you expect them to do? PLEASE TICK ALL THAT APPLY

Answer Options	Response Perce	Response Count
Not to intervene, it is the responsibility of the trial participant's GP and regular healthcare team to monitor and deal with quality of life related disorders such as anxiety and depression, not the trial staff.	24.3 %	27
To discuss the findings with their line manager in the trial, or with the PI.	79.3 %	88
To discuss the findings with a colleague.	8.1 %	9
To discuss the findings with the participant.	24.3 %	27
Using their discretion, arrange an appointment with the patient's GP or other appropriate healthcare professional.	17.1 %	19
Comments		21
	answered ques	111
	skipped questic	18

Q12. Thinking about the future. Please read the following statements. In each case, please indicate whether you 'strongly agree', 'agree', 'have no opinion', 'disagree' or 'strongly disagree' with the statement.

Answer Options	Strongly Agree	Agree	No Opinior	Disagree	Strongly Di	Response (	Strongly Ag	Agree	No Opinior	Disagree	Strongly Di
Data collection staff in trials need more information on Quality of Life/Patient-Reported Outcome measurement - in the trial protocol.	17	33	23	39	3	115	14.8%	28.7%	20.0%	33.9%	2.6%
Data collection staff in trials need more information on Quality of Life/Patient-Reported Outcome measurement - in other trial documentation, such as SOPs.	17	54	19	22	3	115	14.8%	47.0%	16.5%	19.1%	2.6%
Data collection staff in trials need more information on Quality of Life/Patient-Reported Outcome measurement - delivered in the form of trial training.	24	55	17	17	1	114	21.1%	48.2%	14.9%	14.9%	0.9%
There should be specific protocol content and trial training for data collection staff on how to deal with 'concerning' Patient-Reported Outcome information.	27	70	12	5	1	115	23.5%	60.9%	10.4%	4.3%	0.9%
It is important to explain to data collection staff, the purpose and Importance of Quality of Life/Patient-Reported Outcome data to the trial.	41	69	3	0	0	113	36.3%	61.1%	2.7%	0.0%	0.0%
It is important to explain to data collection staff, the relevance and reasoning behind individual Quality of Life/Patient-Reported Outcome questions.	30	55	18	12	0	115	26.1%	47.8%	15.7%	10.4%	0.0%
Comments						14					
					answered (	115					
					skipped qu	14					

Q13. Thinking about the future. What particular Quality of Life/Patient-Reported Outcome guidance should be included the trial protocol, what should be included in trial training, what should be included in a standard operating procedure, and what guidance should not be included in any of the above? PLEASE TICK ALL THAT APPLY

Answer Options	Trial Protocol	Trial Training	Standard C	None	Response (	Trial Proto	Trial Trainir	Standard Operating Procedure
Purpose/Importance of Quality of Life/Patient-Reported Outcome data in trial.	77	89	28	1	115	67.5%	78.1%	24.6%
How to administer the questionnaire.	43	101	73	1	114	38.1%	89.4%	64.6%
When to administer the questionnaire.	88	95	64	1	115	77.2%	83.3%	56.1%
When/how to deal with 'concerning' Quality of Life/Patient-Reported Outcome information.	49	95	77	2	114	43.8%	84.8%	68.8%
What to do if participants write additional information on their questionnaires (or attach a letter).	14	83	78	5	115	12.7%	75.5%	70.9%
Ethical issues associated with Quality of Life/Patient-Reported Outcome use.	57	68	36	12	113	56.4%	67.3%	35.6%
How to deal with upset patients (communication/counselling skills).	6	91	50	14	114	6.0%	91.0%	50.0%
Working with non-English language patients.	39	80	66	9	111	38.2%	78.4%	64.7%
How to support the participant to answer sensitive questions.	4	100	45	6	114	3.7%	92.6%	41.7%
How to collect Quality of Life/Patient-Reported Outcome data without biasing the results.	32	98	63	3	115	28.6%	87.5%	56.3%
Collecting Quality of Life/Patient-Reported Outcome data in different patient groups and/or settings.	24	75	42	18	114	25.0%	78.1%	43.8%
Relevance and reasoning behind individual Quality of Life/Patient-Reported Outcome questions.	50	66	17	23	115	54.3%	71.7%	18.5%
How to deal with difficult situations.	2	88	41	15	114	2.0%	88.9%	41.4%
Comments					4			
				answered (	115			
				skipped qu	14			

Q14. Has completing this questionnaire changed the way you will manage quality of life/patient-reported outcome measurement in future trials?

Answer Options	Response Perce	Response Count
No	56.3 %	63
Yes	43.8 %	49
Comments		30
	answered ques	112
	skipped questic	17

National CTU CI/PI Survey

Q1. First, some questions about yourself. How much experience in total have you had as a CI/PI?

Answer Options	Response P	Response Count
Less than 1 year	0.0 %	0
1-3 years	29.7 %	11
4-6 years	10.8 %	4
7-9 years	13.5 %	5
10 years or more	45.9 %	17
	answered c	37
	skipped qu	0

Q2. Which of the following age groups do you belong to?

Answer Options	Response P	Response Count
25 or younger	0.0 %	0
26-35	13.5 %	5
36-45	29.7 %	11
46-55	37.8 %	14
56 or older	18.9 %	7
	answered c	37
	skipped qu	0

Q3. Thinking about the last trial you worked on that used a Quality of Life or other Patient-Reported Outcome measure. Which of the following clinical areas did the trial cover? PLEASE TICK ALL THAT APPLY

Answer Options	Response P	Response Count
General Practice	33.3 %	9
Orthopaedics	3.7 %	1
General Medicine	0.0 %	0
Rheumatology	18.5 %	5
Cardiovascular	0.0 %	0
Oncology	3.7 %	1
Elderly Care	7.4 %	2
Respiratory	11.1 %	3
Opthalmology	25.9 %	7
Obstetrics & Gynaecology	7.4 %	2
Paediatrics	22.2 %	6
Neurology	14.8 %	4
Comments		16
	answered c	27
	skipped qu	10

Q4. Was the trial based in primary or secondary care?

Answer Options	Response P	Response Count
Primary Care	44.4 %	16
Secondary Care	55.6 %	20
Comments		4
	answered c	36
	skipped qu	1

Q5. In the study, Were you a CI or PI?

Answer Options	Response P	Response Count
CI	60.6 %	20
PI	39.4 %	13
Comments		0
	answered c	33
	skipped qu	4

Q6. Which of the following Patient-Reported Outcome Measures did the trial use? PLEASE TICK ALL THAT APPLY

Answer Options	Response P	Response Count
Euroqol EQ-5D	80.0 %	24
Health Assessment Questionnaire (HAQ)	6.7 %	2
Nottingham Health Profile (NHP)	0.0 %	0
SF-12® Health Survey or SF-12v2™ Health Survey	23.3 %	7
SF-36® Health Survey or SF-36v2™ Health Survey	20.0 %	6
Hospital Anxiety and Depression scale (HAD)	36.7 %	11
Arthritis Impact Measurement Scales (AIMS2)	6.7 %	2
EORTC QLQ - C30 (Core Questionnaire)	0.0 %	0
Minnesota Living with Heart Failure © Questionnaire (MLHF)	3.3 %	1
Oxford Hip Score (OHS)	3.3 %	1
Oxford Knee Score (OKS)	0.0 %	0
Roland-Morris Disability Questionnaire (RMDQ)	13.3 %	4
Don't know	3.3 %	1
Can't remember	6.7 %	2
Comments		21
	answered c	30
	skipped qu	7

Q7. At what stage of the trial design phase was the quality of life/patient-reported outcome element first discussed?

Answer Options	Response P	Response Count
From the start	91.7 %	33
Part way through the process	5.6 %	2
At the end of the process	2.8 %	1
Comments		2
	answered c	36
	skipped qu	1

Q8. Was a quality of life/patient-reported outcomes expert involved in the design phase of the trial?

Answer Options	Response P	Response Count
No	20.6 %	7
Yes	79.4 %	27
Comments		8
	answered c	34
	skipped qu	3

Q9. In your opinion, on a scale of 1 (not important at all) to 10 (extremely important), with what level of importance did the trial management group view the quality of life/patient reported outcome(s) within the trial?

Answer Options	1 (not impo	2	3	4	5	6	7	8	9	10 (extrem	Rating Ave	Response C
	0	0	1	0	0	1	7	2	6	18	8.8	35
Comments												7
											answered c	35
											skipped qu	2

Q10. During the trial, were the staff involved in data collection given instructions on how to administer the quality of life/patient-reported outcome questionnaire?

Answer Options	Response P	Response Count
No	17.1 %	6
Yes	82.9 %	29
Comments		7
	answered c	35
	skipped qu	2

Q11. Again, thinking about the same trial. What particular information on Quality of Life/Patient-Reported Outcome measurement was given to the data collection staff? Please read the option below and in each case select either 'included in trial protocol, training or SOP', or 'not included'.

Answer Options	Included in	Not includ	Response C	Included in	Not included
The purpose and/or Importance of Quality of Life/Patient-Reported Outcome data to the trial.	27	2	29	93.1%	6.9%
Relevance and reasoning behind individual Quality of Life/Patient-Reported Outcome questions.	22	7	29	75.9%	24.1%
When to administer the questionnaire (time points).	29	0	29	100.0%	0.0%
When to administer the questionnaire during the clinic appointment (before/during/after the consultation).	22	6	28	78.6%	21.4%
How much assistance to give the participant during questionnaire completion.	25	4	29	86.2%	13.8%
How to check for, and deal with, missing Quality of Life/Patient-Reported Outcome data.	24	5	29	82.8%	17.2%
How to deal with Quality of Life/Patient-Reported Outcome information that raises concern for the wellbeing of the trial participant (e.g. a questionnaire indicating	22	7	29	75.9%	24.1%
What to do if participants write additional information on their questionnaires (or attach a letter).	12	17	29	41.4%	58.6%
Comments			1		
		answered c	29		
		skipped qu	8		

Q12. Some research nurses we have spoken to have reported encountering Quality of Life/Patient-Reported Outcome questionnaires containing answers which raise concern for the wellbeing of the trial participant in some way. This information has been termed: 'concerning' Patient-Reported Outc

Answer Options	Response P	Response Count
No	50.0 %	18
Yes	50.0 %	18
Don't know	0.0 %	0
	answered c	36
	skipped qu	1

Q13. Have you ever taken action in response to 'concerning' Patient-Reported Outcome information you have encountered/been made aware of within a trial, in order to assist a trial participant?

Answer Options	Response P	Response Count
No	16.7 %	3
Yes	83.3 %	15
Comments		7
	answered c	18
	skipped qu	19

Q14. Was there a mechanism in place to record all action(s) taken in response to the 'concerning' Patient-Reported Outcome information, in the trial documentation?

Answer Options	Response P	Response Count
No	22.2 %	4
Yes	72.2 %	13
Not applicable	5.6 %	1
Comments		3
	answered c	18
	skipped qu	19

Q15. If your data collection staff were to encounter 'concerning' Patient-Reported Outcome information in a future trial, for example, evidence of anxiety or depression, which of the following would you expect them to do? PLEASE TICK ALL THAT APPLY

Answer Options	Response P	Response Count
Not to intervene, it is the responsibility of the trial participant's GP and regular healthcare team to monitor and deal with quality of life related disorders such as a	12.1 %	4
To discuss the findings with their line manager in the trial, or with the PI.	81.8 %	27
To discuss the findings with a colleague.	21.2 %	7
To discuss the findings with the participant.	51.5 %	17
Using their discretion, arrange an appointment with the patient's GP or other appropriate healthcare professional.	30.3 %	10
Comments		7
	answered c	33
	skipped qu	4

Q16. Thinking about the future. Please read the following statements. In each case, please indicate whether you 'strongly agree', 'agree', 'have no opinion', 'disagree' or 'strongly disagree' with the statement.

Answer Options	Strongly Ag	Agree	No Opinion	Disagree	Strongly Di	Response C	Strongly Ag	Agree	No Opinion	Disagree	Strongly Disagree
Data collection staff in trials need more information on Quality of Life/Patient-Reported Outcome measurement - in the trial protocol.	6	12	12	5	1	36	16.7%	33.3%	33.3%	13.9%	2.8%
Data collection staff in trials need more information on Quality of Life/Patient-Reported Outcome measurement - in other trial documentation, such as SOPs.	7	16	8	4	1	36	19.4%	44.4%	22.2%	11.1%	2.8%
Data collection staff in trials need more information on Quality of Life/Patient-Reported Outcome measurement - delivered in the form of trial training.	6	14	7	8	1	36	16.7%	38.9%	19.4%	22.2%	2.8%
There should be specific protocol content and trial training for data collection staff on how to deal with 'concerning' Patient-Reported Outcome information.	14	17	3	2	0	36	38.9%	47.2%	8.3%	5.6%	0.0%
It is important to explain to data collection staff, the purpose and Importance of Quality of Life/Patient-Reported Outcome data to the trial.	12	20	2	1	1	36	33.3%	55.6%	5.6%	2.8%	2.8%
It is important to explain to data collection staff, the relevance and reasoning behind individual Quality of Life/Patient-Reported Outcome questions.	8	22	4	0	2	36	22.2%	61.1%	11.1%	0.0%	5.6%
Comments						6					
					answered c	36					
					skipped qu	1					

Q17. Thinking about the future. What particular Quality of Life/Patient-Reported Outcome guidance should be included the trial protocol, what should be included in trial training, what should be included in a standard operating procedure, and what guidance should not be included in any of the ab

Answer Options	Trial Protoc	Trial Trainii	Standard C	None	Response C	Trial Proto	Trial Trainir	Standard Operating Procedure
Purpose/Importance of Quality of Life/Patient-Reported Outcome data in trial.	27	23	15	1	32	87.1%	74.2%	48.4%
How to administer the questionnaire.	13	27	23	0	32	40.6%	84.4%	71.9%
When to administer the questionnaire.	23	28	21	0	32	71.9%	87.5%	65.6%
When/how to deal with 'concerning' Quality of Life/Patient-Reported Outcome information.	15	27	28	0	32	46.9%	84.4%	87.5%
What to do if participants write additional information on their questionnaires (or attach a letter).	5	25	22	0	32	15.6%	78.1%	68.8%
Ethical issues associated with Quality of Life/Patient-Reported Outcome use.	12	24	16	2	32	40.0%	80.0%	53.3%
How to deal with upset patients (communication/counselling skills).	6	29	17	0	32	18.8%	90.6%	53.1%
Working with non-English language patients.	18	24	20	1	32	58.1%	77.4%	64.5%
How to support the participant to answer sensitive questions.	8	27	19	2	31	27.6%	93.1%	65.5%
How to collect Quality of Life/Patient-Reported Outcome data without biasing the results.	9	29	21	0	32	28.1%	90.6%	65.6%
Collecting Quality of Life/Patient-Reported Outcome data in different patient groups and/or settings.	12	23	16	3	32	41.4%	79.3%	55.2%
Relevance and reasoning behind individual Quality of Life/Patient-Reported Outcome questions.	12	23	10	4	32	42.9%	82.1%	35.7%
How to deal with difficult situations.	5	27	22	2	32	16.7%	90.0%	73.3%
Comments					4			
				answered c	32			
				skipped qu	5			

Q18. Has completing this questionnaire changed the way you will manage quality of life/patient-reported outcome measurement in future trials?

Answer Options	Response P	Response Count
No	73.5 %	25
Yes	26.5 %	9
Comments		6
	answered c	34
	skipped qu	3
